# Gut microbiome mediates the association between dietary quality and metabolic risk in a heterogeneous adult population

**DOI:** 10.1186/s12986-026-01077-5

**Published:** 2026-01-20

**Authors:** Madeline Bartsch, Linda Hemmelrath, Felix Kerlikowsky, Anja Bruns, Milena Burhop, Josefine Nebl, Theresa Greupner, Till Strowig, Till R. Lesker, Lena Amend, Marius Vital, Shoma Berkemeyer, Andreas Hahn, Mattea Müller

**Affiliations:** 1 Institute of Food and One Health, Leibniz University Hannover, 30167 Hannover, Germany; 2 NutritionLab, Faculty of Agricultural Sciences and Landscape Architecture, Osnabrueck University of Applied Sciences, 49090 Osnabrueck, Germany; 3 Institute for Medical Microbiology and Hospital Epidemiology, Hannover Medical School, 30625 Hannover, Germany; 4 Department of Microbial Immune Regulation, Helmholtz Centre for Infection Research, 38124 Braunschweig, Germany; 5 Hannover Medical School, 30625 Hannover, Germany; 6 Centre for Individualized Infection Medicine (CiiM), Helmholtz Centre for Infection Research (HZI), Hannover Medical School (MHH), 30625 Hannover, Germany; 7 Peter L. Reichertz Institute for Medical Informatics, Hannover Medical School, 30625 Hannover, Germany

**Keywords:** Diet quality, Gut microbiome, Metabolic risk

## Abstract

**Background:**

Diet is a determinant of metabolic health, partly through its effects on the gut microbiome, which influences nutrient metabolism, inflammation, and energy balance. We investigated the mediating role of gut microbiome features in the association between dietary quality and metabolic risk.

**Methods:**

In this cross-sectional study, we included 269 adults aged 25–76 years with heterogeneous metabolic profiles, BMI ranging from 17.5 to 47.6 kg/m², and fasting glucose levels between 5.6 and 6.9 mmol/L. Dietary quality was assessed using the Healthy Eating Index (HEI-MON), the Planetary Health Diet Index (PHEI-MON), and the alternate Mediterranean Diet Score (aMED), derived from food-frequency questionnaires and three-day food records. Metabolic risk was quantified using a continuous metabolic syndrome score (cMetS) incorporating waist circumference, mean arterial pressure, HDL cholesterol, triglycerides, and fasting glucose. Microbiome composition (16 S rRNA gene sequencing) and predicted SCFA pathways were analyzed using adjusted multiple linear regression, PERMANOVA, and differential abundance analysis. Mediation analyses examined microbial features as potential mediators of the association between diet and metabolic risk.

**Results:**

Higher HEI-MON, PHEI-MON, and aMED were associated with lower cMetS (*q* < 0.01). *Christensenellaceae R7 group* and *Ruminococcaceae NK4A214 group* were enriched with higher dietary quality and lower cMetS (*q* < 0.1), whereas *Lachnoclostridium* were associated with lower diet quality and higher cMetS (*q* < 0.1). The Enterotype Dysbiosis Score (EDS) correlated inversely with dietary quality (PHEI-MON *q* = 0.04) and positively with cMetS (*q* = 0.04). Butyrate-synthesis pathways were more abundant in individuals with higher dietary quality (*q* < 0.05) and inversely associated with cMetS (*q* < 0.05). Mediation analysis indicated that the *Ruminococcaceae NK4A214 group*,* the Christensenellaceae R7 group*, and *Lachnoclostridium* accounted for up to 16% of the association between diet and metabolic risk.

**Conclusion:**

Better dietary quality is associated with lower metabolic risk and positive gut microbiome signatures across taxonomic, functional, and stability-related aspects. Certain taxa statistically mediated these associations, highlighting gut microbiome features that may contribute to observed links between dietary patterns and metabolic health.

**Graphical abstract:**

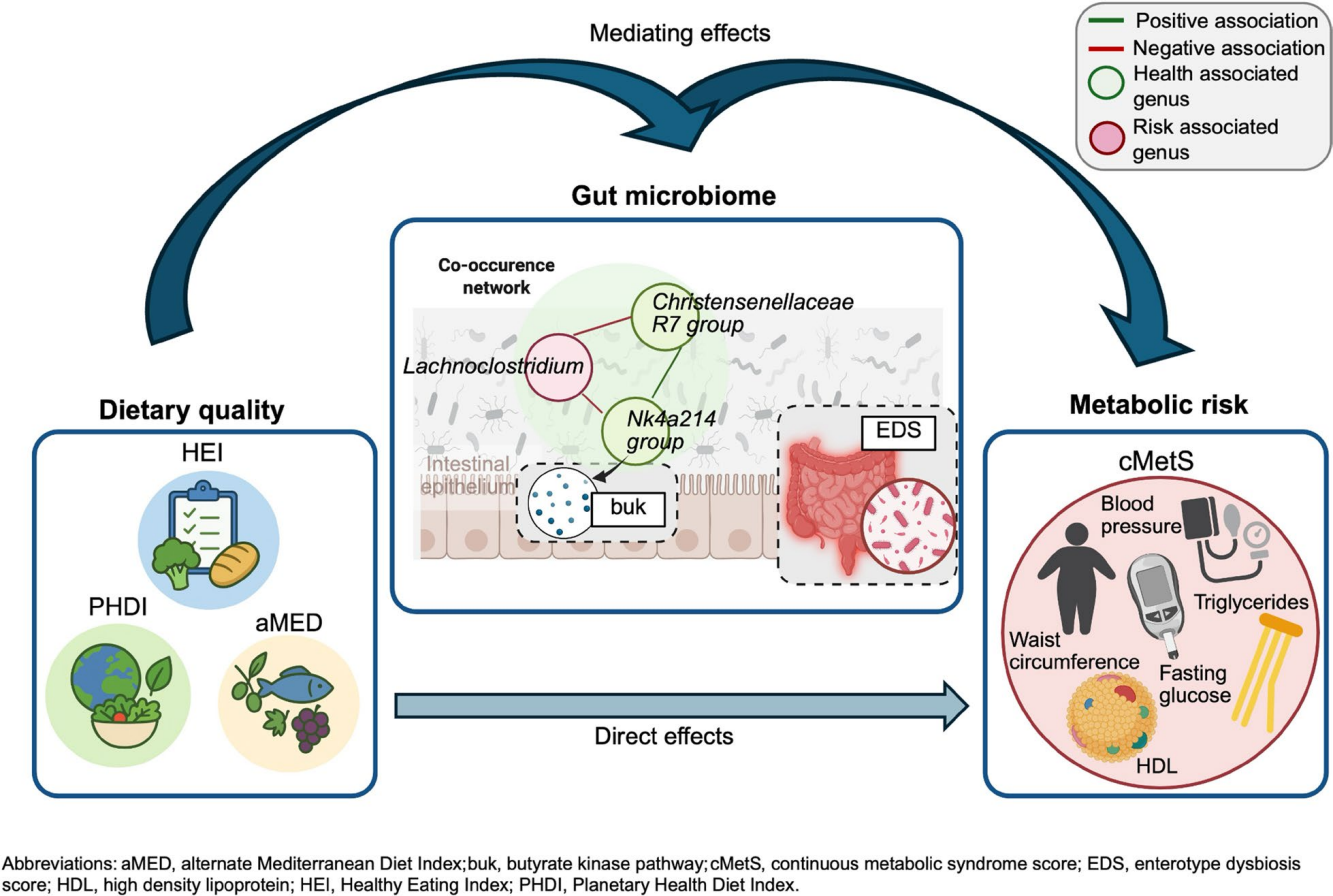

**Supplementary Information:**

The online version contains supplementary material available at 10.1186/s12986-026-01077-5.

## Introduction

Metabolic diseases, including cardiovascular disease, type 2 diabetes mellitus (T2DM), and obesity, represent a substantial global health burden, accounting for a majority of deaths worldwide [1]. Poor diet quality is a key modifiable risk factor, typically characterized by high intakes of sodium, added sugars, and saturated fats, as well as insufficient consumption of fruits, vegetables, and whole grains [2]. While many studies have examined how dietary patterns affect individual metabolic markers such as blood lipids or glucose levels [3–5], single biomarkers often fail to capture the complexity of metabolic dysregulation. Composite measures, such as the continuous metabolic syndrome score (cMetS), which integrates waist circumference (WC), blood pressure, lipid profiles, and glucose metabolism, enable a more comprehensive assessment of metabolic risk [6].

To quantify adherence to health-promoting dietary patterns, several standardized scoring systems have been developed. In this study, we applied three complementary indices: the Healthy Eating Index (HEI-MON), the Planetary Health Diet Index (PHEI-MON), and the alternate Mediterranean Diet Score (aMED). The HEI-MON and PHEI-MON are recent adaptations of the HEI-2020 [7] and Planetary Health Diet Index (PHDI) [8], respectively, for use in German populations based on food frequency questionnaire (FFQ) data [9]. The HEI-MON reflects adherence to current nutritional guidelines, whereas the PHEI-MON incorporates both health-related and environmental aspects, promoting a predominantly plant-based diet with limited intake of animal products, added sugars, and refined grains [10]. The aMED captures adherence to traditionally Mediterranean diets [11], characterized by high intakes of plant-based foods and olive oil, as well as moderate consumption of fish, poultry, and dairy [12, 13]. Higher aMED, HEI, or PHDI scores have consistently been associated with favorable metabolic outcomes, including improved lipid profiles, endothelial function, and reduced inflammation [14–19].

The gut microbiome has emerged as an important factor shaping the relationship between diet and metabolic health. It contributes to host physiology through mechanisms such as energy harvest, immune modulation, and the production of microbial metabolites, including short-chain fatty acids (SCFAs). Diet is a major driver of microbial composition and function [20]. High-fiber, polyphenol-rich diets tend to promote diversity and beneficial taxa, whereas diets rich in saturated fats and refined sugars are often associated with dysbiosis and increased abundance of pro-inflammatory species [21]. A central mechanism linking dietary patterns to metabolic outcomes involves the production of SCFAs by microbial fermentation of dietary fibers. These metabolites, particularly acetate, propionate, and butyrate, play important roles in maintaining gut barrier integrity, modulating immune responses, and improving insulin sensitivity [22]. Higher adherence to the Mediterranean diet has been associated with a greater abundance of SCFA-producing taxa and improvements in lipid and glycemic markers [23]. Since the Planetary Health Diet was introduced relatively recently by the EAT-Lancet Commission in 2019, studies investigating the PHDI in relation to the gut microbiome are still limited. Yet related indices, such as the healthful plant-based diet index, have been associated with increased microbial diversity and decreased abundance of taxa linked to inflammation and adverse lipid profiles [24]. Studies comparing multiple dietary indices, such as the HEI, aMED, and plant-based diet scores, have reported associations with microbial beta-diversity, specific microbial genera [25, 26], and alpha-diversity [26]. Despite integrating microbial and clinical data, most previous studies have not examined whether the gut microbiome acts as a causal mediator between dietary quality and metabolic risk. Moreover, the functional and structural properties of the microbiome remain less well explored than ecological diversity and taxonomic composition. To address these gaps, recent research has incorporated additional microbial metrics, such as enterotypes, which are taxonomically defined community types typically dominated by *Bacteroides* or *Prevotella* and may represent functionally distinct microbial communities with different metabolic properties [27]. Enterotypes have been associated with long-term dietary patterns in multiple populations. Specifically, *Bacteroides*-dominated profiles are often associated with Western-style diets rich in animal protein and saturated fats, whereas *Prevotella*-dominated profiles tend to reflect high-fiber, plant-based diets [28]. Such patterns have been linked to differential metabolic responses, particularly with respect to glycemic control and SCFA production [29]. The *Prevotella/Bacteroides* (PB) ratio serves as a continuous alternative to discrete enterotyping and has been proposed as a marker of fiber fermentation capacity, with higher ratios linked to improved postprandial glucose metabolism and weight loss on high-fiber diets [30, 31]. The Enterotype Dysbiosis Score (EDS) quantifies deviation from established enterotype structures, with higher scores indicating potential dysbiosis and associations with adverse metabolic profiles [32]. In addition, predictive tools enable the estimation of SCFA synthesis pathways from 16 S rRNA gene data, including the succinate and propanediol pathways for propionate production and the acetyl-CoA pathway, with its two terminal enzymes, butyrate transferase and butyrate kinase, that catalyzes the generation of butyrate [33]. While shotgun metagenomic sequencing would enable more precise quantification of these pathways, it remains costly and is therefore often replaced in 16 S-based studies by predictive approaches. Building on these concepts, we investigated associations between dietary quality, gut microbiome characteristics, and metabolic risk in a heterogeneous adult population including individuals with healthy metabolic status, impaired glucose metabolism, and elevated cholesterol levels. We examined how adherence to HEI-MON, PHEI-MON, and aMED was associated with microbial diversity, taxonomic composition, enterotype structure, PB ratio, EDS, and predicted SCFA synthesis pathways. Furthermore, we assessed whether these microbiome features were associated with the cMetS and explored their potential mediating role in the relationship between diet and metabolic health.

## Methods

### Study participants

This cross-sectional analysis included men and women aged 25 to 76 years, with BMI ranging from 17.5 to 47.6 kg/m². Participants were recruited between January 2020 and December 2021 through public advertisements, online announcements, and local media outlets, predominantly in the greater Hannover area of Germany. Eligibility criteria required either elevated low-density lipoprotein cholesterol (LDL: 4.15–5.69 mmol/L), impaired fasting glucose (5.6–6.9 mmol/L) as defined by the American Diabetes Association [34], or overall good health without chronic diseases. Screening involved a standardized health questionnaire, medical history, and anthropometric assessment. Exclusion criteria included severe chronic disease (e.g., diabetes mellitus, cancer, cardiovascular or gastrointestinal disorders), self-reported alcohol, drug, or medication dependence, and current use of immunosuppressive agents or laxatives, as assessed by questionnaires and medication history at study entry. Probiotic use was assessed at baseline, and none of the participants in this cohort reported probiotic use. Written informed consent was obtained from all participants. Of the 296 individuals with available baseline data, seven were excluded for not meeting the inclusion criteria, and 20 were excluded due to missing fecal samples and/or incomplete dietary records, resulting in a final analytical sample of 269 participants (Supplementary Fig. 1).

### Study design and baseline investigation

This secondary analysis is based on baseline data from three previously conducted intervention and cross-sectional studies, registered at the German Clinical Trials Register (DRKS) under DRKS00020384 [35], DRKS00017537 [36], and DRKS00019887 [37]. All baseline investigations were conducted at the Institute of Food and One Health, Leibniz University Hannover (Hannover, Germany). The studies followed identical operating procedures for assessments and bio-sample collection. Before the baseline visit, participants completed a three-day food record (covering two weekdays and one weekend day) and collected a stool sample at home. They were instructed to avoid intense physical activity and alcohol the day before the visit.

On the investigation day, following an overnight fast, anthropometric measurements (height, weight, waist- and hip circumference) and fasting blood samples were obtained. Blood pressure and brachial–ankle pulse wave velocity (PWV) were measured using volume plethysmography (BOSCH & SOHN, Germany) with the Boso ABI-system 100 after a five-minute rest, as previously described [35]. Systolic and diastolic blood pressure were measured bilaterally at the posterior and anterior tibial arteries. PWV was determined based on pulse wave transit time and body height. Participants also completed a validated semi-quantitative FFQ. The studies were approved by the Ethics Committee of the Medical Association of Lower Saxony (Hanover, Germany) and conducted in accordance with the Declaration of Helsinki (October 2008, Seoul).

### Dietary assessment and diet quality indices

Short-term dietary intake was assessed using three-day food records, reviewed for completeness and plausibility by a trained nutritionist. Nutrient intake was analyzed using PRODI expert software (v6.12 and v6.4; Nutri-Science GmbH, Freiburg, Germany). Long-term dietary habits were assessed using the FFQ developed and validated by the Robert Koch Institute, covering 55 food and beverage items, including portion sizes and intake frequencies over the past four weeks [38]. Mean daily intakes in grams were calculated by multiplying the reported intake frequency by the corresponding portion size and dividing the result by 28 days (frequency × portion size in g/28 days) [9].

For alcohol, the average ethanol content of typical serving sizes was calculated (Supplementary Table 1) [39]. For mixed dishes such as hamburgers and doner kebabs, 50% of the portion was allocated to meat. In one study lacking FFQ data, dietary intake was derived from three-day food records by mapping all recorded food items, along with their consumed amounts, to the corresponding FFQ food categories. Based on these quantity-assigned mappings, mean daily intakes (in grams per day) were calculated for each FFQ category, in a manner analogous to the FFQ-based approach, and subsequently used to compute dietary indices.

To assess dietary patterns, we calculated three established indices that reflect different aspects of diet quality. Each item was assigned to index-specific food groups, and scoring was conducted in accordance with the original publications. Higher scores consistently indicated better adherence to the respective pattern. The calculation was implemented in Python (v3.9.6) using custom scripts based on the original scoring algorithms.

#### HEI-MON

The HEI-MON was calculated using the German FFQ and national dietary guidelines, as outlined in [9]. The index comprises ten components: cereals, side dishes, vegetables and legumes, fruits and nuts, milk and dairy products, cheese, meat and processed meat, eggs, fish, and free sugars. Each component was scored on a 0–100 scale based on adherence to intake recommendations and categorized as generous, optimal, or limited. The total HEI-MON score was calculated as the mean of the ten component scores.

#### PHEI-MON

The PHEI-MON was calculated using the same logic and reference framework proposed by Richter et al. (2024), reflecting adherence to the EAT-Lancet planetary health diet [9, 10]. The index comprises 14 components: whole grains, potatoes, vegetables, fruits, legumes, nuts, dairy products, red and processed meats, poultry, eggs, fish, and added sugars. Scoring was based on predefined thresholds reflecting recommended intakes. The overall PHEI-MON score was calculated as the average of all component scores.

#### aMED

The aMED was computed using the algorithm described by Fung et al. (2005) [11]. The score includes nine components: vegetables, legumes, fruits, nuts, whole grains, fish, the monounsaturated-to-saturated fat (MUFA/SFA) ratio, red and processed meat, and alcohol. For beneficial components, one point was assigned if intake exceeded the sample median; for red and processed meat, one point was assigned if intake was below the median. The MUFA/SFA ratio was calculated based on nutrient intake data. Alcohol consumption was evaluated using German-specific cut-offs, assigning one point for < 10 g/day in women and < 25 g/day in men. Wine consumption could not be distinguished from other alcoholic beverages and was included in total alcohol intake. Sex-specific medians were not applied to ensure consistency and comparability across indices.

### Clinical biomarkers and cMetS

Fasting blood samples were collected in EDTA and serum tubes (Sarstedt AG & Co., Nümbrecht, Germany), stored at 4–5 °C, and transferred on the same day to an accredited laboratory (LADR, Hannover, Germany). Lipids and glucose were measured using photometric methods (Beckman Coulter GmbH, Krefeld, Germany). HbA1c was analyzed via high-performance liquid chromatography (Bio-Rad Laboratories GmbH, Feldkirchen, Germany), and insulin was quantified using electrochemiluminescence immunoassay on the cobas e 801 platform (Roche Diagnostics GmbH, Mannheim, Germany). Insulin resistance was estimated using the homeostatic model assessment (HOMA-IR), as previously described [40].

#### cMetS

The cMetS was calculated from WC, mean arterial pressure (MAP), HDL cholesterol, triglycerides, and fasting glucose, as previously described [6]. For each marker, age- and sex-adjusted residuals were obtained from linear regression models and subsequently standardized (z-scores). As HDL cholesterol is inversely associated with metabolic risk, its residuals were multiplied by − 1. The final cMetS score was computed as the sum of all standardized residuals, with higher scores indicating a less favorable metabolic profile.

### Fecal sample collection and gut microbiota sequencing

Participants collected stool at home using kits with RNASepar solution (Biosepar GmbH, Germany) and recorded stool consistency (Bristol Stool Form Scale) (Bristol Stool Form Scale) [41]. Samples were stored at − 80 °C upon arrival. DNA was extracted using the ZymoBIOMICS 96 MagBead DNA Kit (Zymo Research, Germany).

Amplification of the 16 S rRNA V4 region (using primers F515/R806) was performed according to a previously established protocol [42]. DNA was normalized to 25 ng/µl and subjected to PCR using primers containing unique 12-bp Gloray barcodes (Sigma-Aldrich). Each sample was amplified in triplicate with Q5 polymerase (New England Biolabs). The PCR profile consisted of an initial denaturation at 98 °C for 30 s, followed by 25 cycles of 98 °C for 10 s, 55 °C for 20 s, and 72 °C for 20 s. Amplicons were pooled, normalized to 10 nM, and sequenced on an Illumina MiSeq platform with 250 bp paired-end reads. Raw sequences were demultiplexed with idemp (https://github.com/yhwu/idemp) based on the assigned barcodes [42]. Data processing was performed using the USEARCH pipeline (v11.0.667; [43]). Paired reads were merged (fastq_mergepairs; parameters: maxdiffs 30, pctid 70, minmergelen 200, maxmergelen 400), quality-filtered (fastq_filter; maxee 1), dereplicated, and singletons removed (fastx_uniques; minuniquesize 2). Amplicon sequence variants (ASVs/zOTUs) were generated and chimera-checked with unoise3 (parameters: minsize 10, unoise_alpha 2). Amplicon quantification was performed using usearch_global (parameters: strand plus, id 0.97, maxaccepts 10, top_hit_only, maxrejects 250).

Taxonomic classification was conducted using CONSTAX2 [44], which integrates multiple classifiers (rdp, SINTAX, BLAST) against the Greengenes2 reference database [45].

### Statistical analysis

#### Baseline characteristics and associations between diet and cMetS

Sex differences in baseline characteristics were tested using Wilcoxon rank-sum tests. Summary tables were generated using the *gtsummary* package (v2.3.0) [46].

Associations between dietary indices and the cMetS were evaluated using multiple linear regression models. All models included age, sex, and study cohort as covariates. BMI was not included as a covariate in these models because waist circumference is a component of the cMetS, and including BMI would introduce collinearity. For aMED, total energy intake was additionally included, whereas HEI-MON and PHEI-MON were analyzed without energy adjustment because they are kcal-standardized. Standardized beta coefficients with 95% confidence intervals were calculated using the *betaDelta* package (v1.0.5) [47]. All p-values were adjusted for multiple testing using the Benjamini-Hochberg false discovery rate (FDR), with q < 0.05 considered statistically significant.

#### Microbial diversity and community composition

Microbiome data were based on amplicon sequence variant (ASV) abundances processed with the *mia* package (v1.17.5) [48]. Alpha diversity was estimated using Shannon diversity and Faith’s phylogenetic diversity. Associations between dietary indices and alpha diversity were assessed using multiple linear regression models adjusted for age, sex, study cohort, and BMI, with additional adjustment for Bristol stool form and sequencing depth; for aMED models, total energy intake was additionally included as a covariate. Associations between cMetS and alpha diversity were adjusted for age, sex, and study cohort, with additional adjustment for Bristol stool form and sequencing depth.

Beta diversity was calculated using Bray-Curtis dissimilarities of genus-level abundances after CLR transformation with a pseudocount of 1. Redundancy analysis (RDA) and permutational multivariate analysis of variance (PERMANOVA) were performed using *microViz* (v0.12.7) [49] and *adonis2* from the *vegan* package (v2.7–1) [50]. For diet–microbiome analyses, models were adjusted for age, sex, study cohort, BMI, and Bristol stool form. For cMetS–microbiome analyses, models were adjusted for age, sex, study cohort, and Bristol stool form; for aMED models, total energy intake was additionally included as a covariate. FDR-adjusted p-values (Benjamini–Hochberg correction; reported as q-values) < 0.05 were considered statistically significant.

#### Enterotype classification, PB ratio and dysbiosis score

Enterotypes were determined using the web-based EnteroTyper tool (https://enterotype.embl.de/), which applies machine learning algorithms with cross-validation and independent testing [32]. Both two-cluster (*Prevotella* vs. *Bacteroides*/*Phocaeicola*) and three-cluster (*Prevotella*, *Firmicutes*, *Bacteroides*/*Phocaeicola*) solutions were used based on fuzzy k-means and partitioning around medoids (PAM).

To complement enterotype-based classification, the PB ratio was calculated as the natural logarithm of the relative abundance ratio between *Prevotella* and *Bacteroides/Phocaeicola* genera.

Microbial dysbiosis was quantified using the EDS, which reflects the strength and consistency of enterotype assignment across fuzzy clustering iterations. Higher EDS values indicate weaker cluster membership and thus a greater deviation from a typical, well-integrated microbial profile. The score was z-standardized and rescaled to the 0–1 range based on global reference data [32]. Associations between enterotypes, PB ratio, EDS, and dietary indices or cMetS were tested using ANCOVA with Tukey’s post hoc test or linear regression, with the same covariates as in the beta-diversity analysis. All p-values were FDR-corrected, with q < 0.05 indicating significance.

#### Differential abundance analysis (MaAslin3)

Differentially abundant genera were identified using the *MaAslin3* package (v1.1.0) [51]. Genus-level features were filtered for ≥ 10% prevalence and ≥ 1% relative abundance. Differential abundance analyses were performed using MaAsLin3, a multivariable modeling framework specifically developed for microbiome relative-abundance data. Relative abundances were normalized using total sum scaling (TSS) and log-transformed, following the default MaAsLin3 settings [51]. Rather than applying ratio-based transformations such as CLR before modeling, MaAsLin3 models relative abundances directly using a two-part (hurdle) approach that separately evaluates feature presence–absence and variation in non-zero relative abundances. This strategy is designed to address key characteristics of compositional microbiome data, including sparsity, zero inflation, and skewed abundance distributions, while enabling covariate-adjusted association testing within a unified regression framework [51]. As a sensitivity analysis, we additionally reran the MaAsLin3 models using CLR-transformed genus-level data instead of the default TSS + log transformation. Results were consistent in effect direction and statistical significance. Models were fitted separately for each exposure variable, including relevant covariates (see above). Significance was defined as q < 0.1, in accordance with the MaAsLin3 recommendations [51].

#### Correlation between food groups and Microbiome features

Partial Spearman correlations were used to examine associations between food group intake (normalized to 1000 kcal/day) and microbial features, including alpha diversity, predicted SCFA synthesis pathways, and genus-level abundances. CLR-transformed microbiome features were filtered for a prevalence of ≥ 10% and a relative abundance of ≥ 1%. Correlations were adjusted for age and sex using the *pcor.test()* function from the *ppcor* package [52]. Significant associations (q < 0.1) were visualized as annotated heatmaps using *ComplexHeatmap* (v2.25.2) [53].

#### Co-occurrence network analysis

Microbial co-occurrence networks were inferred at the genus-level using the *NetCoMi* package (v1.2.0) [54] in combination with *SpiecEasi* (v1.1.3) [55], applying graphical lasso for network inference. Genus-level features were filtered for prevalence ≥ 10% and relative abundance > 1%, and then CLR-transformed with a pseudocount of 1. Network properties, such as centrality and community clustering (fast greedy algorithm), were calculated using *netAnalyze()*, and networks were visualized with *ggplot2* (v2.2.1) using a Fruchterman–Reingold layout. Co-occurrence network analyses were conducted in an exploratory, hypothesis-generating manner.

#### Prediction of SCFA synthesis pathways

To estimate the microbial potential for SCFA production, we predicted pathway abundances from 16 S rRNA gene data using a customized PICRUSt2 pipeline following Kircher et al. (2022) [33]. This method relies on reference genomes from the Unified Human Gastrointestinal Genome (UHGG) catalog that were screened for SCFA-related biosynthetic pathways using a multilevel strategy. Pathway detection employed hidden Markov models (HMMs) for key enzymes, synteny analysis, and evaluation of pathway completeness. Butyrate formation was assessed via the acetyl-CoA pathway, with its two terminal enzymes, butyrate transferase (but) and butyrate kinase (buk). Propionate formation was captured through the propanediol (pdiol) and succinate (succite) pathways. We utilized the publicly available reference files (https://github.com/ag-vital/predict_SCFA_producers), which included a curated reference tree, aligned pathway sequences, and trait abundance tables. ASVs from our dataset were placed into the reference phylogeny using PICRUSt2’s place_seqs.py, and predicted pathway presence per ASV was obtained using hsp.py with the empirical probability model. Results were then summed across all ASVs per sample and normalized to relative abundances. Associations with dietary indices and the cMetS were analyzed using multiple linear regression, with the covariates described above.

#### Mediation analysis

To assess whether gut microbiota features mediated associations between dietary quality and metabolic health, we performed causal mediation analyses using the *mediation* package (v 4.5.1) [56]. Separate models were run for each dietary index, with microbial parameters as mediators and cMetS as outcome. Mediators included alpha diversity, PB ratio, EDS, predicted SCFA pathways, and genera retained after prevalence and abundance filtering. All models were adjusted using the same covariate structure as in the primary analyses. Average causal mediation effects (ACME), average direct effects (ADE), total effects, and proportion mediated were estimated using quasi-Bayesian Monte Carlo approximation with 1,000 simulations. P-values for ACME were adjusted for Multiple Comparisons across all mediators per exposure, with *q* < 0.05 indicating significance. For individual taxa, a Bonferroni correction (q < 0.10) was applied to account for the larger number of tests.

Across all analyses in this study, multiple testing correction was applied separately within each analytical framework and study question (e.g., diet–cMetS associations, diversity analyses, differential abundance analyses, and mediation analyses), rather than pooling all p-values across the entire study. All statistical analyses were performed in R (v4.5.1).

## Results

### Participant characteristics and dietary quality

A total of 269 participants were included in the final analyses (Supplementary Fig. 1). The median age was 57 years, with women being older than men (61 vs. 49 years; *p* < 0.01). Overall, 62% of participants were female (*n* = 168) and 38% were male (*n* = 101). The median BMI was 25.5 kg/m². Sex-specific differences appeared in several clinical parameters: women had significantly higher levels of total, HDL, and LDL cholesterol (all *p* < 0.01) and higher fasting insulin levels (*p* = 0.02), while men had slightly higher systolic blood pressure (*p* = 0.02) and women had slightly higher diastolic blood pressure (*p* = 0.04) (Supplementary Table 2).

**Fig. 1:**
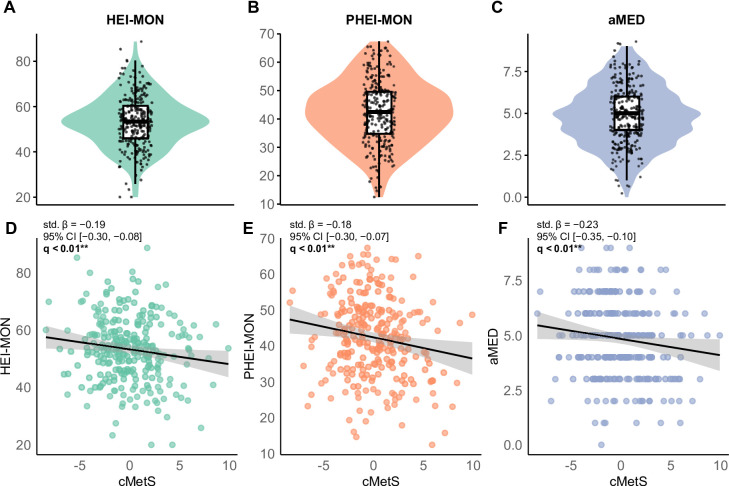
Dietary quality scores and their associations with metabolic health. Panels A to C display the distributions of three dietary indices: (**A**) Healthy Eating Index adapted to German dietary guidelines (HEI-MON), (**B**) Planetary Health Eating Index adapted to the Planetary Health Diet (PHEI-MON), and (**C**) alternate Mediterranean Diet Score (aMED). Violin plots illustrate full distributions, with boxplots indicating medians and interquartile ranges, and individual values are overlaid. Panels D–F show associations between each index and the continuous metabolic syndrome score (cMetS): (**D**) HEI-MON, (**E**) PHEI-MON, and (**F**) aMED. Scatterplots depict individual data points with linear regression lines and 95% confidence intervals. Models were adjusted for age, sex, and study cohort; energy intake was additionally included as a covariate in the aMED model. P-values were corrected for multiple testing using the false discovery rate (FDR), with q < 0.05 considered statistically significant

Median dietary index scores were 53 (IQR 46–60) for HEI-MON, 42 (IQR 35–50) for PHEI-MON, and 5 (IQR 4–6) for aMED (Fig. 1A–C), with no significant differences between sexes. All three indices were negatively associated with cMetS (all *q* < 0.01; Fig. 1D–F), with standardized coefficients of β = − 0.19 for HEI-MON, β = − 0.18 for PHEI-MON, and β = − 0.23 for aMED.

### Microbiome data characteristics

The median sequencing depth was 20,174 reads per sample (IQR 15,009–30,103). A total of 3,305 ASVs, representing 345 genera across 19 bacterial phyla, were detected across all samples. Alpha- and beta-diversity analyses were conducted using the full, unfiltered genus-level dataset. For taxa-based analyses, including differential abundance testing, food group correlations, co-occurrence network analysis, and mediation analyses, genus-level features were filtered to include only those with a prevalence ≥ 10% and a relative abundance ≥ 1%. Following this filtering step, 45 genera were retained for downstream analyses. Across all samples, the median Shannon diversity was 3.97 (IQR 3.58–4.22), and the median Faith’s phylogenetic diversity was 7.00 (IQR 6.03–8.02).

### Diet quality is associated with beta, but not with alpha diversity

**Fig. 2:**
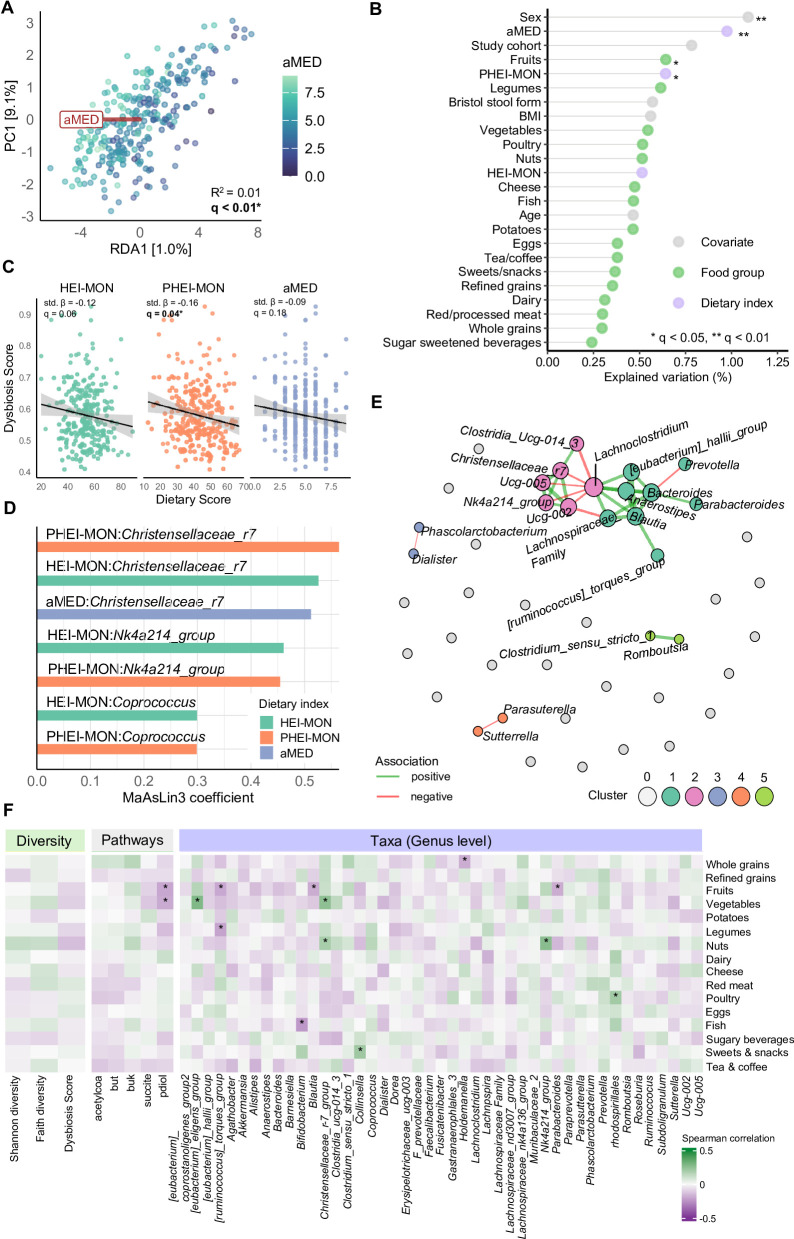
Associations between dietary patterns and gut microbiota composition and structure. (**A**) Redundancy analysis (RDA) plot showing genus-level, CLR-transformed gut microbial composition constrained by the alternate Mediterranean Diet Score (aMED). Each point represents an individual, colored by aMED. (**B**) Proportion of variance in microbial beta diversity explained by dietary indices and selected food groups, based on PERMANOVA using Bray–Curtis dissimilarity. Models were adjusted for age, sex, BMI, study cohort, and stool consistency; for aMED models, total energy intake was additionally included as a covariate. Asterisks indicate significance after FDR correction (*q < 0.05, **q < 0.01). (**C**) Associations between dietary scores (HEI-MON, PHEI-MON, aMED) and gut microbial dysbiosis score. Models were adjusted as in (**B**). (**D**) Significant genus-level associations identified by MaAsLin3 for the three dietary indices. Bars indicate standardized regression coefficients. Taxa were filtered based on ≥ 10% prevalence and ≥ 1% relative abundance. Associations with FDR-adjusted q < 0.1 were considered significant. (**E**) Genus-level co-occurrence network inferred using SpiecEasi (graphical lasso) and visualized with a Fruchterman–Reingold layout. Nodes represent genera, scaled by eigenvector centrality and colored by cluster. Edges indicate conditional associations; line width and color reflect strength and direction (green = positive, red = negative). (**F**) Heatmap of partial Spearman correlations between dietary intake (per 1000 kcal) and microbial features: diversity metrics (left), SCFA-associated pathways (center), and genus-level taxa (right). Correlations were adjusted for age and sex. *q < 0.1

To examine the relationship between microbial diversity and dietary patterns, both alpha- and beta-diversity were analyzed. No significant links were found between any of the dietary indices and microbial alpha-diversity (all *q* > 0.05; Supplementary Fig. 2). However, the aMED score accounted for the largest proportion of variance in microbial composition (R² = 0.01, *q* < 0.01; Fig. 2A–B), followed by PHEI-MON (R² = 0.006, *q* = 0.01), while HEI-MON showed a weaker, non-significant trend (R² = 0.005, *q* = 0.06; Supplementary Fig. 3).

### No strong microbial enterotype signatures of diet quality

To explore community-level structure, participants were classified into microbial enterotypes. A two-cluster solution (*Bacteroides*/*Phocaeicola* vs. *Prevotella*) assigned 146 individuals to the first cluster and 123 to the second. A three-cluster solution resulted in 46 *Bacteroides*-, 152 *Firmicutes*-, and 70 *Prevotella*-type assignments. None of the dietary indices differed significantly between clusters in either solution (all *q* > 0.30; Supplementary Fig. 4A–G). Similarly, the PB ratio was not associated with any dietary index, although a trend toward association with aMED was observed (*q* = 0.08; Supplementary Fig. 5A–C). In contrast, the EDS showed an inverse association with PHEI-MON (std. β = − 0.16, *q* = 0.04), indicating that higher adherence to this dietary pattern was linked to lower EDS, while no statistically significant associations were observed for HEI-MON (*q* = 0.06) or aMED (*q* = 0.18) (Fig. 2C).

### Genus-level associations with dietary indices and food groups

To identify genera associated with diet quality, genus-level differential abundance analyses were conducted. All three dietary indices were positively associated with the relative abundance of *Christensenellaceae R-7 group* (HEI-MON: β = 0.53, *q* = 0.02; PHEI-MON: β = 0.56, *q* = 0.01; aMED: β = 0.51, *q* = 0.05). A higher abundance of *NK4A214_group* was associated with a higher HEI-MON (β = 0.46, *q* = 0.02) and PHEI-MON (β = 0.45, *q* = 0.05) score. *Coprococcus* was also positively associated with HEI-MON (β = 0.30, *q* = 0.07) and PHEI-MON (β = 0.30, *q* = 0.09) (Fig. 2D).

To investigate potential contributors to these associations, Spearman correlations between genus-level abundances and energy-adjusted food group intakes were calculated, and supported the observed associations. To investigate whether specific food groups contributed to these associations, Spearman correlations were calculated between genus-level relative abundances and energy-adjusted intakes of food groups. Vegetable consumption was positively correlated with the abundance of *Eubacterium_eligens_group* (ρ = 0.28, *q* < 0.01) and *Christensenellaceae R-7 group* (ρ = 0.25, *q* = 0.01). Legume intake showed an inverse correlation with *Ruminococcus torques group* (ρ = − 0.21, *q* = 0.07). Nut consumption was positively associated with *Ruminococcaceae NK4A214_group* (ρ = 0.26, *q* < 0.01), and poultry intake correlated positively with a genus assigned to *Rhodospirillales* (ρ = 0.21, *q* = 0.09). In contrast, fish intake was negatively associated with *Bifidobacterium* (ρ = − 0.24, *q* = 0.02), and consumption of sweets and snacks showed a positive correlation with *Collinsella* (ρ = 0.21, *q* = 0.07) (Fig. 2F).

### Microbial co-occurrence networks reveal key community structures

To explore whether microbial genera associated with dietary quality co-occur in patterns that potentially indicate functionally related clusters, we constructed a genus-level correlation network. The resulting network was partitioned into five microbial co-occurrence modules, representing groups of genera with strong internal connectivity and relatively few links to other genera, as identified by greedy modularity optimization. One module showed a negative association between *Sutterella* and *Parasutterella*, whereas another showed a positive co-occurrence between *Romboutsia* and *Clostridium sensu stricto.* A third, smaller module showed a negative connection between *Phascolarctobacterium* and *Dialister*. Two larger modules dominated the network: the first centered on *Lachnoclostridium* and featured negative associations with *Clostridia UCG-014*, *Ruminococcaceae UCG-002*, *Ruminococcaceae NK4A214*, and *Ruminococcaceae UCG-005*. A highly connected subcluster comprised *Christensenellaceae R-7*,* Ruminococcaceae UCG-005*, and *NK4A214.* The second central module was organized around *Bacteroides*, which showed a negative association with *Prevotella* and featured positive associations with *Anaerostipes*, the *Eubacterium hallii* group, *Parabacteroides*, *Blautia*, and *Ruminococcus torques* group (Fig. 2E).

### Microbial SCFA biosynthesis potential reflects dietary patterns

To assess functional differences in microbial SCFA metabolism, predicted pathway abundances associated with SCFA biosynthesis were analyzed in relation to diet quality indices. All indices showed significant positive associations with the predicted *buk* pathway (HEI-MON: std. β = 0.26, *q* < 0.001; PHEI-MON: std. β = 0.25, *q* < 0.001; aMED: std. β = 0.21, *q* < 0.01). PHEI-MON was also positively associated with the *acetyl-CoA* and *but* pathways (std. β = 0.18, *q* = 0.02 and std. β = 0.16, *q* = 0.04, respectively). The *pdiol* pathway was inversely associated with aMED (std. β = −0.17, *q* = 0.02), but showed no association with HEI-MON or PHEI-MON. No significant associations were found for the succinate pathway (q > 0.05) (Supplementary Fig. 6). Genera contributing to the *buk* and acetyl-CoA pathways also overlapped with taxa identified in the differential abundance analysis (e.g., *Coprococcus*, *Ruminococcaceae NK4A214*; Supplementary Table 4).

### Gut microbial features are associated with metabolic health

**Fig. 3:**
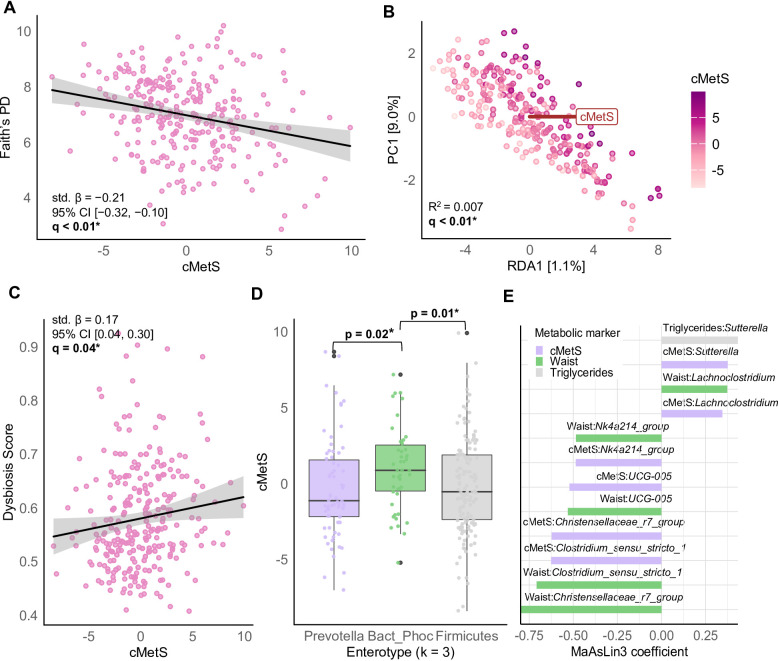
Associations between gut microbiome composition and metabolic risk. (**A**) Negative association between Faith’s phylogenetic diversity and the continuous metabolic syndrome score (cMetS). Models were adjusted for age, sex, study cohort, stool consistency, and sequencing depth. (**B**) Redundancy analysis (RDA) plot showing genus-level, CLR-transformed microbial composition constrained by cMetS. Each point represents one individual, colored by cMetS value. PERMANOVA models were adjusted for age, sex, study cohort, and stool consistency. (**C**) Differences in cMetS across genus-level enterotypes (k = 3). Statistical comparison was performed using ANCOVA adjusted for age, sex, study cohort, and stool consistency. Pairwise comparisons were tested using Tukey’s post hoc test. (**D**) Positive association between the dysbiosis score and cMetS. Models were adjusted for age, sex, study cohort, and stool consistency. (**E**) Significant associations between microbial genera and metabolic markers identified by MaAsLin3. Bars indicate MaAsLin3 regression coefficients. Taxa were filtered based on ≥ 10% prevalence and ≥ 1% relative abundance. Models were adjusted for age, sex, study cohort, and stool consistency. Associations with FDR-adjusted q < 0.1 were considered statistically significant

Metabolic health, as reflected by the cMetS score, was significantly associated with multiple dimensions of gut microbial composition and diversity. Individuals with higher cMetS scores showed significantly lower phylogenetic alpha-diversity, as indicated by an inverse association with Faith’s phylogenetic diversity (std. β = −0.21, *q* < 0.01; Fig. 3A). Consistently, cMetS was also associated with differences in overall microbiota composition, with a significant association in beta-diversity analyses (R² = 0.007, *q* < 0.01; Fig. 3B). Furthermore, we examined cMetS in relation to community structure defined by enterotype clustering. In the two-cluster solution, no statistically significant differences in cMetS were observed (*q* = 0.38; Supplementary Fig. 3 C). However, the three-cluster solution revealed notable differences: individuals classified into the *Bacteroides*/*Phocaeicola*-dominant enterotype had significantly higher cMetS scores compared to those in the *Firmicutes*-dominant (*p* = 0.01) and *Prevotella*-dominant clusters (*p* = 0.02; Fig. 3C). Beyond community types, the dysbiosis score was positively associated with metabolic risk. Higher cMetS scores correlated with a higher dysbiosis score (std. β = 0.17, *q* = 0.04; Fig. 3D), whereas no significant association was found for the PB ratio (*q* = 0.21; Supplementary Fig. 4D). Differential abundance analysis identified several microbial taxa associated with cMetS. Higher scores were linked to a lower relative abundance of *Christensenellaceae R-7 group* (β = −0.62, *q* < 0.01), *UCG-005* (β = −0.52, *q* < 0.01), *Clostridium sensu stricto 1* (β = −0.63, *q* < 0.01), and *Ruminococcaceae NK4A214 group* (β = −0.49, *q* = 0.03). In contrast, *Lachnoclostridium* (β = 0.35, *q* = 0.02) and *Sutterella* (β = 0.37, *q* = 0.04) were more abundant in individuals with higher metabolic risk (Fig. 3E).

To further examine whether the associations identified for the composite cMetS were driven by the overall metabolic risk or by specific components, we performed an additional differential abundance analysis using the five individual MetS components as predictors. Significant associations with gut microbial taxa were observed only for WC and triglyceride levels (Fig. 3E). Specifically, WC was inversely associated with *Christensenellaceae R-7 group* (β = −0.80, *q* < 0.001), *Ruminococcaceae UCG-005* (β = −0.48, *q* = 0.04), *Clostridium sensu stricto 1* (β = −0.71, *q* = 0.01), and *Ruminococcaceae NK4A214 group* (β = −0.52, *q* = 0.05), while *Lachnoclostridium* was more abundant in individuals with higher WC (β = 0.37, *q* = 0.07). Triglyceride levels were positively associated with *Sutterella* (β = 0.43, *q* = 0.05). No significant associations were found for fasting glucose, HDL-cholesterol, or blood pressure,

Finally, the potential for microbial SCFA production in relation to metabolic health was examined by analyzing predicted pathway abundances. Among all pathways, only the *buk* pathway showed a significant association with cMetS, with higher scores correlating with a lower relative abundance of *buk* (std. β = −0.21, *q* < 0.01; Supplementary Fig. 5). Notably, *Ruminococcaceae NK4A214 group* and *Ruminococcaceae UCG-005*, both negatively associated with cMetS, were among the main contributors to the *buk* pathway in the dataset (Supplementary Table 4).

### Microbial mediation: microbiota as a potential link between diet and metabolic health

**Fig. 4:**
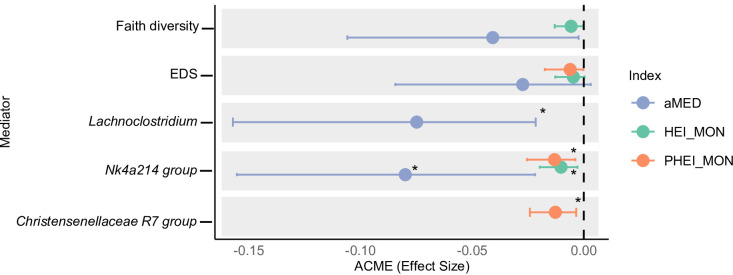
Mediation analysis: Microbiota as potential mediator of dietary patterns and metabolic risk. Average causal mediation effects (ACME; mediated effect sizes) with 95% confidence intervals for significant mediators in the association between dietary indices (HEI-MON, PHEI-MON, aMED) and metabolic risk (cMetS). Points represent ACME point estimates, and horizontal lines indicate 95% CIs. Mediation models were adjusted for age, sex, stool consistency, study cohort, and energy intake. All p-values were FDR-adjusted. Overall mediation effects were considered significant at *q* < 0.05. For individual taxa, a Bonferroni correction (*q* < 0.10) was applied to account for the larger number of tests. Only variables with ACME *q* < 0.10 are shown. EDS, enterotype dysbiosis score

To investigate whether the associations between diet quality and metabolic health were mediated by gut microbial features, mediation analyses were performed, with cMetS as the outcome and each dietary index (HEI-MON, PHEI-MON, and aMED) as the exposure. Microbiome-related mediators included alpha-diversity metrics, the PB ratio, the EDS, predicted SCFA biosynthesis pathways, and genera that were differentially abundant in response to diet. No significant mediation effects were observed in overall alpha diversity. However, Faith’s phylogenetic diversity showed trends toward mediation for both HEI-MON (ACME = − 0.006, *q* = 0.09; proportion mediated = 11.22%) and aMED (ACME = − 0.04, *q* = 0.09; proportion mediated = 7.19%). The EDS showed similar trends across all three dietary indices, mediating 8.54% of the effect of HEI-MON (ACME = − 0.005, *q* = 0.09), 10.73% for PHEI-MON (ACME = − 0.009, *q* = 0.09), and 5.01% for aMED (ACME = − 0.027, *q* = 0.09) (Fig. 4). The PB ratio did not mediate the association between any dietary index and cMetS (all *q* > 0.10).

Among the predicted SCFA pathways, the unadjusted models suggested a potential mediating role for the *buk* pathway across all three scores (all *p* < 0.05). However, none of these effects remained significant after FDR correction (all *q* > 0.10).

At the genus level, the *Ruminococcaceae NK4A214 group* significantly mediated the associations between cMetS and all three dietary indices (HEI-MON: ACME = − 0.02, *q* = 0.01; proportion mediated = 13.58%; PHEI-MON: ACME = − 0.01, *q* = 0.01; 15.10%; aMED: ACME = − 0.08, *q* < 0.01; 10.20%) (Fig. 4).

*Lachnoclostridium* emerged as a significant mediator of the aMED–cMetS association (ACME = − 0.07, *q* < 0.01; proportion mediated = 14.02%), and *Christensenellaceae R-7 group* mediated the association between PHEI-MON and cMetS (ACME = − 0.01, *q* = 0.09; 16.89%)z (Fig. 4).

## Discussion

In this study, we examined the relationships between dietary quality, gut microbiome characteristics, and metabolic health in a heterogeneous adult population. Across three complementary dietary indices, higher adherence was associated with lower metabolic risk, as measured by the cMetS, and with distinct taxonomic, functional, and ecological microbiome features. The *Ruminococcaceae NK4A214 group* emerged as a consistent statistical mediator of the diet–metabolic health link across all dietary indices. In contrast, the *Christensenellaceae R7 group* mediated the association with PHEI-MON, and *Lachnoclostridium* mediated the association with aMED. Co-occurrence network analysis revealed that health-associated genera tended to cluster together, with some risk-associated genera, such as *Lachnoclostridium*, occurring in the same module but connected through negative edges. The EDS captured diet–microbiome–metabolic associations more sensitively than enterotype classifications or PB ratio. Functional predictions indicated higher abundances of butyrate-producing pathways, particularly the *buk* and *acetyl-CoA* routes, in participants with higher dietary quality and lower metabolic risk, with *Ruminococcaceae NK4A214 group* contributing to these functions.

The inverse association between dietary quality and metabolic risk aligns with evidence that adherence to health-promoting dietary patterns, including Mediterranean-like and plant-based diets, improves cardiometabolic outcomes [14, 17–19] through combined effects of fiber-rich foods, phytochemicals, favorable fatty acid profiles, and reduced intake of refined carbohydrates and processed meats [57–61]. While such relationships are well established, our study contributes to this body of evidence by identifying specific microbial taxa and pathways that may explain some of the observed benefits of a high-quality diet.

The identification of *Ruminococcaceae NK4A214 group* as mediating genus across all dietary indices highlights its potential importance for metabolic health. This taxon was relatively more abundant in participants with healthier diets and lower cMetS scores. Notably, these relationships were observed irrespective of whether diet quality was defined by Mediterranean pattern, national nutritional guidelines, or the Planetary Health Diet, suggesting that *Ruminococcaceae NK4A214 group* responds to overall healthy dietary patterns rather than to the specific composition of any single index. Previous research has linked this genus to improved insulin sensitivity [62], greater weight loss success [63], and enhanced systemic recovery after stroke [64], supporting a role in metabolic resilience through SCFA production, improved gut barrier function, and modulation of inflammatory pathways [65, 66]. Nuts and vegetables were the strongest dietary contributors in our cohort, likely due to fermentable fibers and polyphenols that selectively promote this genus [67]. Although thresholds for “low” abundance have not yet been established, its position within a statistical mediation framework linking diet quality and metabolic health suggests that abundance could serve as a marker of a metabolically resilient microbiome and as a target for dietary modulation.

*Christensenellaceae R7 group* showed similar beneficial associations but mediated only the association between PHEI-MON and cMetS, perhaps reflecting differences in the scoring of plant-based components such as whole grains, legumes, and nuts. Higher intakes of plant-derived components, particularly vegetables, dietary fiber, and polyphenols, have been linked to increased abundance of *Christensenellaceae* [68], consistent with our food–group–specific observations. This genus is inversely associated with markers of metabolic dysfunction, such as BMI, visceral adiposity, liver fat, HOMA-IR, circulating triglycerides, and total, LDL, and HDL cholesterol [69]. It is known to produce acetate, which can support butyrate-producing microbes through cross-feeding [70] and influence host lipid metabolism, potentially contributing to the observed links with improved metabolic profiles [22].

As co-occurrence network analyses are exploratory and descriptive, the *Christensenellaceae R7* group clustered with *Ruminococcaceae NK4A214* and other health-associated taxa, a configuration that may reflect previously described functional interdependencies within the gut microbial community [71]. The positive connections among these taxa suggest a cooperative consortium in which metabolic cross-feeding, such as the provision of acetate by *Christensenellaceae* that *Ruminococcaceae* can use for butyrate synthesis, may reflect functional interdependencies previously described, including SCFA-related cross-feeding and gut barrier–related processes, as well as metabolic regulation [71]. Similar positive associations between *Christensenellaceae* and *Ruminococcaceae* have also been reported in another network analysis of a comparable study population, supporting the robustness of this ecological association [72]. *Lachnoclostridium*, in contrast, was positioned within the same module but showed negative correlations with members of this beneficial cluster. Such inverse connections may indicate competitive or antagonistic relationships described in the literature, although no causal or functional inferences can be drawn from co-occurrence patterns alone [71]. Such patterns suggest that dietary patterns associated with beneficial clusters may also be linked to a lower abundance of risk-associated taxa [73].

*Lachnoclostridium* was more abundant in individuals with higher metabolic risk and mediated the association between aMED and cMetS. Higher aMED scores were associated with lower *Lachnoclostridium* abundance. Unlike HEI-MON and PHEI-MIN, aMED emphasizes fat quality, particularly a higher monounsaturated-to-saturated fat ratio, which may explain why the association is observed specifically for this index. A high monounsaturated-to-saturated fat ratio could modulate bile acid composition and microbial metabolic niches in ways that reduce the competitiveness of *Lachnoclostridium* while supporting SCFA-producing taxa [74]. Moreover, *Lachnoclostridium* encodes pathways for trimethylamine and cytidine diphosphate-diacylglycerol biosynthesis, both of which are implicated in cardiometabolic dysfunction [75], supporting its potential characterization as a risk-associated taxon linked to adverse metabolic profiles.

Beyond taxonomic associations, functional pathway predictions provided additional insights, with higher dietary quality and lower metabolic risk associated with greater abundances of butyrate-producing pathways, particularly the buk and acetyl-CoA routes [33], to which *Ruminococcaceae NK4A214 group* contributed substantially. These findings are supported by previous intervention studies, which have reported increases in fecal butyrate concentrations following a Mediterranean diet intervention [76]. Butyrate plays a well-established role in maintaining intestinal barrier integrity [77], reducing systemic inflammation, and enhancing insulin sensitivity [78]. Interestingly, the acetyl-CoA pathway was specifically associated with PHEI-MON, which may better reflect substrate availability for this pathway, given its explicit emphasis on diverse plant-based foods. Not all SCFA-related pathways aligned with beneficial profiles. The *pdiol* pathway, involved in microbial propionate production from fucose, rhamnose, and polyols [79], was inversely associated with aMED scores and fruit/vegetable intake. While propionate can be beneficial, intermediates such as 1,2-propanediol may have pro-inflammatory effects under certain conditions and can serve as substrates for pathobionts, including *Salmonella* and adherent-invasive *Escherichia* coli, thereby facilitating their expansion in dysbiotic gut environments [53, 80]. Therefore, a lower abundance of this pathway in individuals adhering to Mediterranean-like diets may reflect reduced functional niches for pathobionts.

Despite prior research linking dietary patterns to enterotype clustering [28], we found no associations between diet quality and either two- or three-cluster enterotypes or PB ratio. These findings may reflect the influence of host, lifestyle, or environmental factors, or the limited resolution of both categorical enterotypes and the PB ratio for detecting subtle diet–microbiome links when using dietary indices.

Associations with metabolic status were more pronounced among individuals with the *Bacteroides*/*Phocaeicola*-dominant enterotype, which was more common among those with higher metabolic risk, consistent with previous reports that this enterotype is associated with lower microbial diversity and a more proinflammatory profile. However, such observations are not consistently reported across studies, suggesting that this enterotype is not inherently detrimental but may be context-dependent [81].

The EDS provided a more nuanced perspective. This continuous metric, developed to quantify taxonomic imbalance relative to a metabolically healthy reference, was significantly associated with both the PHDI and the cMetS in our dataset. Higher EDS values have been linked to diseases such as colorectal cancer, Crohn’s disease, and ulcerative colitis [32], and in our cohort, they coincided with lower diet quality and greater metabolic risk. This suggests that composite measures of ecological imbalance may capture microbiome–host relationships more sensitively than fixed community-type classifications, particularly in smaller cohorts or when enterotype separation is weak.

Our findings suggest that integrating dietary indices with taxonomic, functional, and structural microbiome measures can provide a more comprehensive understanding of the pathways linking diet and metabolic health. The identification of the *Ruminococcaceae NK4A214 group* as a consistent mediator across dietary patterns, together with the *Christensenellaceae R7 group* and *Lachnoclostridium* as diet-specific mediators embedded in shared co-occurrence clusters, suggests that not only individual taxa but also their ecological interactions may be relevant to understanding the observed associations. These features, along with the added sensitivity of the EDS, highlight promising microbiome targets for advancing personalized nutrition strategies. Future longitudinal and intervention studies should examine whether targeted dietary modifications can increase the abundance of beneficial taxa, strengthen health-associated microbial networks, and reduce dysbiosis scores, and whether such changes translate into measurable improvements in metabolic health. If confirmed, these microbiome features could be incorporated into risk assessment frameworks and dietary guidelines, moving the field closer to microbiome-informed precision nutrition.

Several limitations should be considered when interpreting these findings. Dietary intake was assessed using FFQs and three-day food records, both of which are prone to recall bias and measurement error. The cross-sectional design precludes definitive conclusions about causality or the temporal sequence between diet, microbiome features, and metabolic risk. Although individuals with diagnosed chronic diseases were excluded, the presence of undiagnosed or pre-clinical disease states cannot be ruled out and may have influenced metabolic or microbiome-related measures. In addition, information on smoking status and physical activity was unavailable and therefore could not be included as covariates, which may have resulted in residual confounding of the observed associations. While our mediation analysis provides insight into possible causal pathways, longitudinal or interventional data are needed to confirm these relationships. Microbiome characterization was based on 16 S rRNA gene sequencing, which limits taxonomic resolution to the genus level. Although we complemented this with predictive functional profiling to explore microbial metabolic pathways, such predictions are inherently uncertain and cannot replace direct metagenomic or metabolomic measurements. The sample size may have reduced statistical power to detect weaker associations or mediators. In addition, despite adjusting for sex in all models, the unequal sex distribution in the study population may have limited the ability to detect potential sex effect modification, particularly for microbiome-related outcomes that are sensitive to sample size. Future studies with larger, more balanced cohorts are needed to robustly assess sex-specific associations.

Furthermore, WC was used as a surrogate for visceral adiposity, and no biomarkers of adipose tissue function or systemic inflammation were available to explore mechanistic pathways in greater depth. Finally, our findings are based on the cMetS, calculated as the sum of age- and sex-adjusted residuals of individual MetS components. Alternative severity scores, such as the Gurka et al. score derived from confirmatory factor analysis, were not associated with dietary indices or microbiome features in our cohort. This discrepancy may reflect differences in the constructions of scores. Whereas the severity score applies predefined factor loadings, the residual-z approach allows components to contribute more directly to overall risk. Given that waist circumference emerged as the strongest driver of the observed associations, the cMetS may be particularly sensitive for detecting such cross-sectional relationships in our population.

Overall, higher dietary quality was linked to lower metabolic risk and a gut microbiome profile characterized by beneficial taxa, enhanced SCFA pathways, and cooperative microbial networks. Our findings underscore the value of combining taxonomic, functional, and ecological metrics to capture diet–microbiome–metabolism interactions. These features represent promising features for future investigation in dietary intervention studies and may support the development of microbiome-informed strategies to prevent and manage metabolic disease.

## Supplementary Information

Supplementary Material 1

## Data Availability

Custom R and Python scripts used in this study have been deposited in a public repository under [https://github.com/madelinebar/diet_microbiome_analysis]. Due to privacy regulations, individual-level metadata cannot be made publicly available but can be obtained from the corresponding author upon reasonable request. Raw 16 S rRNA gene sequencing data have been deposited in the European Nucleotide Archive (ENA) under the accession numbers PRJEB97794.

## Abbreviations

aMED: Alternate Mediterranean Diet Score
ACME: Average causal mediation effects
ADE: Average direct effects
ASVs: Amplicon sequence variants
BMI: Body mass index
Buk: Butyrate kinase
but butyryl-CoA: Acetate CoA-transferase
cMetS: Continuous metabolic syndrome score
EDS: Enterotype dysbiosis score
FFQ: Food Frequency Questionnaire
FDR: False discovery rate
HEI-MON: Healthy Eating Index
HMMs: Hidden Markov models
LDL: Low-density lipoprotein
MUFA/SFA: Monounsaturated-to-saturated fat ratio
OTUs: Operational taxonomic units
PB ratio: Prevotella/Bacteroides ratio
PERMANOVA: Permutational multivariate analysis of variance
PHEI-MON: Planetary Health Diet Index
PWVL: Pulse wave velocity
Pdiol: Propanediol pathway
RDA: Redundancy analysis
SCFAs: Short-chain fatty acids
Succite: Succinate pathway
T2DM: Type 2 diabetes mellitus
TSS: Total sum scaling
UHGG: Unified Human Gastrointestinal Genome
WC: Waist circumference
