# Supplementary material for: Gut microbiome mediates the association between dietary quality and metabolic risk in a heterogeneous adult population
Source: Nutr Metab (Lond). 2026 Jan 20;23:13. doi: 10.1186/s12986-026-01077-5 (PMC12849433; doi:10.1186/s12986-026-01077-5)
Supplement: Supplementary file 1 — Supplementary Material 1 [file 12986_2026_1077_MOESM1_ESM.docx]

**Supplementary Table 1: Estimated alcohol content per beverage category**

| **Beverage category** | | **Estimated alcohol content [% vol]** |
| --- | --- | --- |
| Beer | 4 | |
| Wine, sparkling wine | 9 | |
| Cocktails | 6.5 | |
| Spirits | 32 | |
| Median alcohol contents (% vol) for beer, wine/sparkling wine, cocktails, and spirits used in dietary intake calculations. Values are based on typical beverage data reported in Wahrburg and Egert (2015). | | |

**Supplementary Table 2: Summary characteristics of participants**

| **Characteristic** | **Overall**  (n = 269) | **Male**  **(**n = 101) | **Female**  (n = 168) | **p-value** |  |
| --- | --- | --- | --- | --- | --- |
| **Main Characteristics** |  |  |  |  |  |
| **Age (y)** | **57 (36, 66)** | **49 (33, 64)** | **61 (43, 67)** | **<0.01** |  |
| BMI (kg/m²) | 25.5 (22.8, 28.7) | 25.2 (23.3, 27.9) | 25.9 (22.1, 29.4) | >0.90 |  |
| **Waist-Hip Ratio** | **0.84 (0.77, 0.90)** | **0.89 (0.81, 0.96)** | **0.81 (0.76, 0.87)** | **<0.001** |  |
| **Systolic BP (mmHg)** | **134 (125, 146)** | **137 (129, 147)** | **131 (122, 146)** | **0.02** |  |
| **Diastolic BP (mmHg)** | **83 (76, 90)** | **81 (75, 88)** | **85 (76, 91)** | **0.04** |  |
| **PWV (m/s)** | **7.00 (6.00, 8.20)** | **6.70 (5.90, 7.90)** | **7.45 (6.10, 8.60)** | **<0.01** |  |
|  |  |  |  |  |  |
| **Metabolic Biomarkers** |  |  |  |  |  |
| **Total Cholesterol (mmol/L)** | **5.85 (4.50, 7.19)** | **5.25 (4.10, 6.75)** | **6.29 (4.85, 7.34)** | **<0.001** |  |
| **HDL (mmol/L)** | **1.58 (1.30, 1.81)** | **1.44 (1.11, 1.59)** | **1.68 (1.40, 1.91)** | **<0.001** |  |
| **LDL (mmol/L)** | **4.02 (2.71, 4.85)** | **3.74 (2.57, 4.65)** | **4.26 (3.06, 4.95)** | **<0.01** |  |
| TG (mmol/L) | 1.16 (0.83, 1.61) | 1.03 (0.81, 1.58) | 1.19 (0.87, 1.63) | 0.20 |  |
| Glucose (mmol/L) | 5.50 (5.00, 5.80) | 5.50 (5.10, 5.80) | 5.40 (5.00, 5.80) | 0.40 |  |
| HbA1c^†^ (%) | 5.20 (5.00, 5.54) | 5.20 (5.00, 5.54) | 5.20 (5.00, 5.54) | >0.90 |  |
| **Insulin^†^ (µU/mL)** | **7.40 (5.20, 12.2)** | **5.90 (4.70, 10.1)** | **8.50 (5.50, 12.9)** | **0.02** |  |
| HOMA-IR^†^ | 1.70 (1.06, 2.84) | 1.32 (0.96, 2.60) | 2.05 (1.20, 3.20) | 0.05 |  |
| MetS | -0.20 (-2.30, 2.10) | -0.30 (-2.10, 2.10) | -0.20 (-2.10, 1.90) | 0.60 |  |
| **Diet Quality Indices** |  |  |  |  |  |
| HEI-MON | 53 (46, 60) | 53 (46, 59) | 53 (46, 62) | 0.40 |  |
| PHEI-MON | 42 (35, 50) | 41 (32, 49) | 43 (35, 50) | 0.10 |  |
| aMED | 5 (4, 6) | 5 (3, 6) | 5 (4, 6) | 0.30 |  |
| Median values and interquartile ranges (Q1, Q3) are shown for all participants (n = 269) and stratified by sex. Group comparisons were performed using the Wilcoxon rank-sum test. Variables include anthropometric measures, metabolic biomarkers, and dietary indices. Data on dietary indices and select biomarkers were available for n = 226. Abbreviations: aMED, alternate Mediterranean Diet Score; BP, blood pressure; HDL, high-density lipoprotein; HEI-MON, Healthy Eating Index (based on the German Nutrition Society’s FBDG); HOMA-IR, homeostasis model assessment of insulin resistance; LDL, low-density lipoprotein; MetS, metabolic syndrome (Z-score based on waist circumference, triglycerides, HDL cholesterol, glucose, and mean arterial pressure); PHEI-MON, Planetary Health Eating Index (based on the EAT-Lancet diet adapted to German dietary patterns); PWV, pulse wave velocity; TG, triglycerides. | | | | | |

**Supplementary Table 3: Spearman correlations between energy-adjusted intake of selected food groups and relative abundance of bacterial genera**

|  | Whole grains | | Refined grains | | Fruits | | Vegetables | | Potatoes | | Legumes | | Nuts | | Dairy | | Cheese | | Red & processed meat | | Poultry | | Eggs | | Fish | | Sugary beverages | | Sweets & snacks | | Tea & coffee | |  |
| --- | --- | --- | --- | --- | --- | --- | --- | --- | --- | --- | --- | --- | --- | --- | --- | --- | --- | --- | --- | --- | --- | --- | --- | --- | --- | --- | --- | --- | --- | --- | --- | --- | --- |
|  | ρ | adj. P | ρ | adj. P | ρ | adj. P | ρ | adj. P | ρ | adj. P | ρ | adj. P | ρ | adj. P | ρ | adj. P | ρ | adj. P | ρ | adj. P | ρ | adj. P | ρ | adj. P | ρ | adj. P | ρ | adj. P | ρ | adj. P | ρ | adj. P |  |
| shannon_diversity | 0.01 | 0.95 | -0.10 | 0.65 | 0.00 | 0.98 | 0.02 | 0.93 | -0.01 | 0.95 | 0.02 | 0.93 | 0.12 | 0.52 | 0.05 | 0.82 | 0.02 | 0.94 | -0.08 | 0.70 | -0.05 | 0.85 | -0.04 | 0.85 | 0.04 | 0.88 | -0.06 | 0.80 | -0.02 | 0.94 | -0.02 | 0.93 |  |
| faith_diversity | 0.04 | 0.85 | 0.04 | 0.85 | 0.02 | 0.93 | 0.10 | 0.64 | -0.08 | 0.68 | 0.02 | 0.93 | 0.11 | 0.57 | 0.00 | 1.00 | 0.09 | 0.68 | 0.09 | 0.65 | -0.04 | 0.87 | -0.03 | 0.89 | 0.07 | 0.79 | -0.08 | 0.68 | 0.00 | 0.99 | 0.07 | 0.76 |  |
| Enterotype_Dysbiosis_Score_k3 | -0.01 | 0.96 | -0.02 | 0.94 | -0.08 | 0.68 | -0.10 | 0.65 | -0.01 | 0.97 | -0.08 | 0.68 | -0.14 | 0.41 | -0.02 | 0.93 | -0.04 | 0.86 | 0.07 | 0.80 | 0.04 | 0.87 | 0.06 | 0.81 | -0.03 | 0.88 | -0.04 | 0.87 | 0.02 | 0.93 | 0.00 | 0.98 |  |
| [eubacterium]_coprostanoligenes_group_2 | -0.01 | 0.97 | 0.06 | 0.80 | -0.07 | 0.76 | 0.03 | 0.91 | 0.04 | 0.85 | -0.04 | 0.86 | -0.02 | 0.94 | -0.02 | 0.94 | -0.01 | 0.97 | -0.02 | 0.94 | 0.03 | 0.91 | -0.08 | 0.68 | -0.04 | 0.87 | 0.01 | 0.95 | 0.04 | 0.86 | 0.00 | 0.97 |  |
| [eubacterium]_eligens_group | 0.11 | 0.57 | -0.10 | 0.61 | 0.16 | 0.30 | 0.28 | 0.00 | -0.01 | 0.95 | -0.03 | 0.93 | 0.04 | 0.85 | -0.02 | 0.93 | 0.12 | 0.56 | -0.08 | 0.68 | -0.06 | 0.81 | 0.00 | 1.00 | 0.09 | 0.68 | -0.01 | 0.95 | -0.08 | 0.68 | 0.01 | 0.95 |  |
| [eubacterium]_hallii_group | -0.06 | 0.80 | -0.04 | 0.85 | -0.10 | 0.59 | -0.10 | 0.65 | 0.06 | 0.82 | -0.01 | 0.95 | -0.10 | 0.59 | -0.02 | 0.94 | -0.09 | 0.67 | -0.09 | 0.66 | 0.01 | 0.95 | 0.08 | 0.68 | -0.05 | 0.83 | 0.08 | 0.68 | 0.01 | 0.97 | -0.05 | 0.82 |  |
| [ruminococcus]_torques_group | -0.13 | 0.45 | 0.03 | 0.91 | -0.20 | 0.12 | -0.14 | 0.43 | -0.11 | 0.57 | -0.21 | 0.07 | -0.12 | 0.56 | 0.10 | 0.59 | 0.08 | 0.70 | 0.13 | 0.48 | 0.05 | 0.82 | 0.10 | 0.59 | 0.06 | 0.81 | 0.00 | 1.00 | -0.02 | 0.93 | -0.10 | 0.65 |  |
| Agathobacter | -0.03 | 0.91 | 0.05 | 0.85 | -0.11 | 0.57 | -0.11 | 0.57 | 0.11 | 0.59 | 0.06 | 0.80 | -0.03 | 0.91 | -0.05 | 0.85 | -0.15 | 0.39 | 0.05 | 0.82 | -0.06 | 0.82 | 0.00 | 0.97 | -0.06 | 0.81 | 0.06 | 0.81 | 0.00 | 0.98 | -0.15 | 0.33 |  |
| Akkermansia | -0.09 | 0.67 | -0.12 | 0.56 | -0.04 | 0.88 | -0.08 | 0.70 | -0.04 | 0.85 | -0.06 | 0.82 | -0.01 | 0.95 | -0.02 | 0.94 | -0.05 | 0.83 | -0.05 | 0.85 | 0.01 | 0.96 | 0.01 | 0.95 | -0.01 | 0.94 | -0.08 | 0.68 | -0.02 | 0.94 | 0.01 | 0.95 |  |
| Alistipes | -0.05 | 0.82 | -0.02 | 0.94 | -0.14 | 0.39 | -0.09 | 0.68 | -0.05 | 0.85 | -0.01 | 0.97 | 0.01 | 0.95 | -0.08 | 0.70 | 0.01 | 0.97 | -0.08 | 0.70 | -0.03 | 0.92 | -0.02 | 0.94 | -0.02 | 0.94 | 0.10 | 0.59 | -0.07 | 0.80 | -0.17 | 0.27 |  |
| Anaerostipes | 0.00 | 0.98 | -0.07 | 0.80 | -0.08 | 0.68 | 0.01 | 0.95 | 0.07 | 0.77 | 0.00 | 0.99 | -0.06 | 0.82 | -0.07 | 0.80 | -0.13 | 0.45 | -0.14 | 0.39 | -0.09 | 0.68 | -0.03 | 0.91 | -0.10 | 0.59 | 0.01 | 0.95 | 0.02 | 0.93 | 0.01 | 0.96 |  |
| Bacteroides | -0.03 | 0.92 | -0.04 | 0.87 | -0.14 | 0.45 | -0.08 | 0.70 | 0.12 | 0.56 | -0.02 | 0.94 | -0.09 | 0.65 | 0.02 | 0.94 | -0.06 | 0.80 | 0.00 | 0.98 | -0.01 | 0.97 | 0.00 | 1.00 | -0.04 | 0.85 | 0.06 | 0.81 | 0.01 | 0.97 | -0.13 | 0.45 |  |
| Barnesiella | -0.06 | 0.80 | -0.06 | 0.82 | -0.11 | 0.57 | -0.06 | 0.81 | -0.06 | 0.81 | -0.07 | 0.78 | -0.04 | 0.87 | 0.07 | 0.77 | 0.02 | 0.93 | -0.01 | 0.97 | 0.05 | 0.82 | 0.01 | 0.94 | -0.02 | 0.94 | 0.04 | 0.86 | 0.01 | 0.94 | -0.06 | 0.82 |  |
| Bifidobacterium | -0.04 | 0.87 | 0.10 | 0.59 | 0.01 | 0.95 | -0.09 | 0.68 | 0.08 | 0.68 | 0.02 | 0.93 | -0.09 | 0.68 | -0.06 | 0.80 | -0.09 | 0.65 | -0.05 | 0.84 | -0.05 | 0.83 | -0.06 | 0.82 | -0.24 | 0.02 | 0.02 | 0.93 | 0.17 | 0.26 | -0.04 | 0.87 |  |
| Blautia | -0.05 | 0.82 | -0.09 | 0.65 | -0.18 | 0.15 | -0.11 | 0.57 | -0.01 | 0.97 | -0.08 | 0.68 | -0.07 | 0.80 | 0.05 | 0.82 | -0.08 | 0.68 | -0.02 | 0.93 | 0.02 | 0.93 | -0.01 | 0.95 | -0.03 | 0.90 | -0.04 | 0.85 | -0.03 | 0.91 | -0.07 | 0.71 |  |
| Christensenellaceae_r-7_group | 0.12 | 0.56 | 0.12 | 0.56 | 0.11 | 0.57 | 0.25 | 0.01 | 0.01 | 0.95 | 0.12 | 0.56 | 0.19 | 0.15 | -0.02 | 0.94 | 0.09 | 0.68 | -0.07 | 0.74 | -0.12 | 0.56 | -0.10 | 0.59 | 0.04 | 0.85 | -0.15 | 0.39 | -0.02 | 0.93 | 0.07 | 0.77 |  |
| Clostridia_ucg-014_3 | 0.02 | 0.93 | 0.00 | 0.98 | 0.04 | 0.87 | 0.14 | 0.45 | -0.03 | 0.91 | 0.04 | 0.85 | 0.12 | 0.55 | -0.09 | 0.68 | -0.08 | 0.68 | -0.11 | 0.57 | -0.10 | 0.59 | -0.11 | 0.57 | -0.05 | 0.85 | -0.17 | 0.27 | -0.02 | 0.93 | 0.12 | 0.53 |  |
| Clostridium_sensu_stricto_1 | 0.16 | 0.29 | -0.05 | 0.85 | 0.13 | 0.45 | -0.04 | 0.87 | -0.04 | 0.87 | -0.03 | 0.91 | 0.08 | 0.68 | 0.00 | 1.00 | 0.02 | 0.93 | -0.01 | 0.97 | -0.13 | 0.45 | -0.04 | 0.87 | 0.05 | 0.83 | 0.02 | 0.93 | 0.05 | 0.85 | -0.12 | 0.56 |  |
| Collinsella | 0.07 | 0.79 | 0.07 | 0.80 | -0.15 | 0.37 | -0.11 | 0.57 | 0.01 | 0.94 | -0.08 | 0.68 | -0.14 | 0.39 | 0.02 | 0.93 | -0.01 | 0.97 | 0.05 | 0.83 | 0.04 | 0.87 | -0.05 | 0.83 | 0.00 | 1.00 | 0.10 | 0.65 | 0.21 | 0.07 | 0.02 | 0.94 |  |
| Coprococcus | 0.07 | 0.78 | 0.07 | 0.80 | 0.01 | 0.95 | 0.04 | 0.87 | -0.03 | 0.88 | 0.13 | 0.48 | 0.15 | 0.39 | 0.08 | 0.70 | -0.02 | 0.94 | -0.12 | 0.52 | -0.01 | 0.97 | 0.05 | 0.85 | 0.09 | 0.67 | 0.05 | 0.82 | 0.01 | 0.96 | 0.04 | 0.86 |  |
| Dialister | -0.02 | 0.93 | -0.09 | 0.68 | 0.03 | 0.93 | -0.16 | 0.29 | -0.01 | 0.96 | 0.00 | 1.00 | 0.03 | 0.91 | -0.03 | 0.90 | -0.16 | 0.29 | -0.03 | 0.91 | 0.01 | 0.97 | -0.11 | 0.58 | -0.14 | 0.45 | 0.03 | 0.91 | -0.03 | 0.91 | -0.03 | 0.91 |  |
| Dorea | -0.11 | 0.59 | 0.04 | 0.87 | -0.13 | 0.48 | -0.11 | 0.57 | -0.06 | 0.82 | -0.14 | 0.39 | -0.08 | 0.68 | 0.06 | 0.80 | 0.00 | 1.00 | 0.05 | 0.85 | 0.02 | 0.94 | -0.02 | 0.94 | 0.00 | 1.00 | 0.08 | 0.68 | 0.03 | 0.88 | -0.04 | 0.88 |  |
| Erysipelotrichaceae_ucg-003 | -0.09 | 0.65 | -0.03 | 0.90 | -0.04 | 0.87 | 0.03 | 0.91 | -0.03 | 0.91 | -0.12 | 0.56 | -0.05 | 0.82 | 0.04 | 0.87 | -0.01 | 0.95 | 0.01 | 0.95 | -0.04 | 0.87 | 0.03 | 0.91 | -0.02 | 0.94 | 0.08 | 0.70 | -0.06 | 0.80 | -0.03 | 0.91 |  |
| F__prevotellaceae | -0.02 | 0.93 | 0.01 | 0.96 | -0.02 | 0.93 | -0.09 | 0.68 | 0.03 | 0.88 | -0.06 | 0.82 | -0.08 | 0.70 | -0.05 | 0.82 | 0.03 | 0.91 | 0.07 | 0.77 | 0.04 | 0.87 | -0.07 | 0.75 | -0.02 | 0.93 | 0.05 | 0.82 | 0.02 | 0.94 | 0.07 | 0.76 |  |
| Faecalibacterium | 0.10 | 0.65 | -0.07 | 0.79 | -0.06 | 0.80 | 0.02 | 0.94 | 0.06 | 0.80 | -0.01 | 0.96 | -0.11 | 0.57 | -0.03 | 0.90 | -0.10 | 0.59 | -0.04 | 0.87 | -0.05 | 0.83 | 0.01 | 0.95 | -0.09 | 0.68 | -0.04 | 0.87 | -0.06 | 0.82 | -0.04 | 0.85 |  |
| Fusicatenibacter | -0.10 | 0.61 | -0.01 | 0.95 | -0.07 | 0.73 | -0.05 | 0.83 | -0.06 | 0.82 | -0.03 | 0.92 | 0.03 | 0.89 | -0.02 | 0.93 | -0.07 | 0.74 | -0.07 | 0.78 | -0.10 | 0.65 | -0.03 | 0.88 | -0.08 | 0.70 | 0.04 | 0.85 | -0.13 | 0.48 | 0.00 | 0.99 |  |
| Gastranaerophilales_3 | -0.05 | 0.83 | -0.08 | 0.68 | 0.01 | 0.95 | 0.04 | 0.87 | -0.05 | 0.85 | 0.02 | 0.93 | 0.03 | 0.91 | 0.08 | 0.68 | 0.01 | 0.95 | -0.10 | 0.65 | 0.13 | 0.45 | -0.04 | 0.87 | 0.05 | 0.82 | -0.05 | 0.85 | -0.08 | 0.68 | 0.05 | 0.82 |  |
| Holdemanella | -0.19 | 0.15 | 0.07 | 0.76 | -0.06 | 0.82 | 0.02 | 0.94 | 0.01 | 0.96 | 0.04 | 0.85 | -0.05 | 0.85 | -0.10 | 0.59 | 0.12 | 0.56 | 0.04 | 0.85 | 0.00 | 1.00 | -0.04 | 0.87 | -0.09 | 0.68 | 0.03 | 0.91 | 0.02 | 0.93 | -0.04 | 0.87 |  |
| Lachnoclostridium | -0.05 | 0.85 | -0.01 | 0.95 | -0.10 | 0.64 | -0.11 | 0.57 | 0.09 | 0.65 | 0.02 | 0.94 | -0.16 | 0.27 | 0.02 | 0.94 | -0.03 | 0.91 | 0.05 | 0.83 | 0.10 | 0.59 | 0.02 | 0.93 | -0.05 | 0.83 | 0.04 | 0.87 | -0.07 | 0.77 | -0.12 | 0.56 |  |
| Lachnospira | -0.02 | 0.94 | -0.03 | 0.88 | 0.06 | 0.81 | 0.02 | 0.93 | 0.06 | 0.82 | 0.13 | 0.47 | 0.04 | 0.87 | -0.17 | 0.24 | -0.09 | 0.65 | -0.10 | 0.65 | -0.13 | 0.48 | -0.04 | 0.87 | -0.02 | 0.94 | 0.08 | 0.68 | -0.03 | 0.91 | -0.12 | 0.52 |  |
| Lachnospiraceae Family | 0.02 | 0.94 | -0.10 | 0.61 | -0.08 | 0.68 | -0.06 | 0.81 | 0.02 | 0.94 | -0.08 | 0.68 | 0.01 | 0.97 | 0.01 | 0.95 | 0.02 | 0.94 | -0.01 | 0.95 | -0.02 | 0.93 | 0.01 | 0.95 | 0.01 | 0.97 | 0.01 | 0.95 | -0.04 | 0.87 | -0.10 | 0.61 |  |
| Lachnospiraceae_nd3007_group | 0.02 | 0.94 | -0.02 | 0.93 | 0.06 | 0.80 | 0.08 | 0.68 | -0.06 | 0.82 | 0.05 | 0.82 | 0.03 | 0.91 | -0.05 | 0.82 | -0.03 | 0.90 | -0.11 | 0.57 | -0.16 | 0.27 | -0.03 | 0.92 | -0.03 | 0.91 | -0.06 | 0.82 | -0.13 | 0.45 | 0.06 | 0.82 |  |
| Lachnospiraceae_nk4a136_group | 0.13 | 0.45 | 0.05 | 0.85 | 0.08 | 0.70 | 0.16 | 0.31 | 0.02 | 0.94 | 0.06 | 0.82 | -0.03 | 0.91 | -0.01 | 0.95 | 0.08 | 0.68 | 0.02 | 0.93 | 0.06 | 0.81 | 0.02 | 0.93 | 0.06 | 0.82 | -0.03 | 0.91 | -0.03 | 0.91 | -0.02 | 0.93 |  |
| Muribaculaceae_2 | 0.00 | 1.00 | 0.01 | 0.95 | 0.00 | 0.98 | -0.08 | 0.68 | 0.08 | 0.68 | 0.03 | 0.91 | 0.02 | 0.94 | 0.01 | 0.95 | 0.06 | 0.81 | 0.07 | 0.76 | 0.05 | 0.83 | 0.05 | 0.83 | 0.03 | 0.91 | 0.04 | 0.87 | 0.06 | 0.80 | -0.03 | 0.91 |  |
| Nk4a214_group | 0.08 | 0.68 | -0.01 | 0.96 | 0.15 | 0.39 | 0.04 | 0.88 | -0.08 | 0.68 | 0.04 | 0.86 | 0.26 | 0.01 | -0.05 | 0.82 | -0.02 | 0.93 | -0.11 | 0.59 | -0.08 | 0.68 | -0.10 | 0.65 | 0.08 | 0.68 | -0.12 | 0.56 | -0.01 | 0.95 | 0.06 | 0.82 |  |
| Parabacteroides | -0.04 | 0.85 | -0.06 | 0.81 | -0.19 | 0.15 | -0.05 | 0.83 | 0.11 | 0.57 | 0.03 | 0.88 | -0.16 | 0.29 | 0.00 | 1.00 | 0.06 | 0.81 | -0.01 | 0.95 | 0.09 | 0.67 | -0.04 | 0.87 | -0.04 | 0.87 | 0.00 | 0.99 | 0.05 | 0.82 | -0.07 | 0.80 |  |
| Paraprevotella | 0.08 | 0.69 | -0.08 | 0.70 | 0.05 | 0.82 | 0.01 | 0.95 | -0.03 | 0.90 | -0.04 | 0.88 | -0.07 | 0.71 | 0.02 | 0.93 | -0.05 | 0.82 | -0.05 | 0.85 | 0.05 | 0.85 | 0.06 | 0.81 | -0.07 | 0.77 | 0.05 | 0.82 | -0.06 | 0.82 | 0.08 | 0.68 |  |
| Parasutterella | -0.04 | 0.85 | 0.09 | 0.65 | 0.02 | 0.93 | 0.08 | 0.68 | 0.08 | 0.68 | -0.02 | 0.93 | 0.12 | 0.56 | 0.04 | 0.85 | 0.02 | 0.93 | 0.06 | 0.81 | 0.01 | 0.97 | 0.09 | 0.68 | 0.02 | 0.94 | 0.02 | 0.93 | -0.04 | 0.88 | -0.03 | 0.91 |  |
| Phascolarctobacterium | -0.04 | 0.85 | 0.09 | 0.68 | -0.06 | 0.82 | 0.06 | 0.80 | 0.02 | 0.93 | -0.02 | 0.94 | -0.13 | 0.48 | -0.06 | 0.81 | 0.05 | 0.83 | 0.13 | 0.45 | -0.01 | 0.95 | 0.04 | 0.85 | 0.00 | 1.00 | -0.03 | 0.88 | 0.02 | 0.93 | -0.02 | 0.93 |  |
| Prevotella | 0.01 | 0.96 | 0.14 | 0.40 | 0.12 | 0.56 | 0.13 | 0.45 | 0.06 | 0.82 | 0.03 | 0.88 | -0.03 | 0.92 | 0.00 | 0.97 | 0.03 | 0.91 | 0.13 | 0.45 | 0.10 | 0.59 | 0.08 | 0.68 | 0.04 | 0.85 | 0.04 | 0.85 | 0.05 | 0.82 | 0.10 | 0.59 |  |
| rhodospirillales | 0.07 | 0.79 | -0.07 | 0.71 | 0.03 | 0.90 | -0.01 | 0.95 | -0.06 | 0.80 | -0.08 | 0.68 | 0.02 | 0.93 | 0.17 | 0.26 | 0.12 | 0.56 | 0.03 | 0.93 | 0.21 | 0.09 | 0.09 | 0.65 | 0.17 | 0.27 | -0.05 | 0.82 | -0.02 | 0.93 | 0.00 | 1.00 |  |
| Romboutsia | 0.01 | 0.95 | -0.04 | 0.87 | -0.09 | 0.65 | -0.11 | 0.59 | -0.02 | 0.93 | -0.08 | 0.68 | 0.06 | 0.82 | -0.03 | 0.92 | -0.06 | 0.81 | 0.07 | 0.79 | -0.04 | 0.87 | 0.03 | 0.91 | -0.05 | 0.82 | 0.06 | 0.82 | 0.04 | 0.85 | -0.04 | 0.85 |  |
| Roseburia | -0.01 | 0.97 | 0.01 | 0.96 | -0.04 | 0.87 | 0.01 | 0.95 | 0.02 | 0.94 | -0.02 | 0.93 | 0.05 | 0.83 | -0.08 | 0.70 | -0.01 | 0.97 | -0.02 | 0.93 | -0.07 | 0.77 | -0.07 | 0.72 | 0.00 | 0.98 | -0.08 | 0.68 | -0.16 | 0.29 | -0.05 | 0.85 |  |
| Ruminococcus | -0.04 | 0.88 | 0.09 | 0.68 | -0.09 | 0.68 | -0.06 | 0.81 | 0.04 | 0.87 | 0.02 | 0.94 | 0.05 | 0.82 | -0.01 | 0.97 | -0.01 | 0.95 | 0.03 | 0.88 | -0.06 | 0.82 | 0.09 | 0.66 | 0.01 | 0.97 | 0.06 | 0.80 | -0.01 | 0.95 | -0.05 | 0.85 |  |
| Subdoligranulum | 0.02 | 0.94 | 0.03 | 0.91 | -0.06 | 0.82 | -0.05 | 0.82 | 0.03 | 0.90 | -0.02 | 0.93 | 0.07 | 0.80 | 0.03 | 0.91 | -0.11 | 0.57 | -0.04 | 0.87 | -0.10 | 0.64 | 0.05 | 0.83 | 0.01 | 0.97 | 0.10 | 0.59 | -0.04 | 0.87 | 0.03 | 0.91 |  |
| Sutterella | -0.04 | 0.87 | -0.08 | 0.68 | -0.05 | 0.83 | -0.16 | 0.28 | -0.06 | 0.81 | 0.02 | 0.93 | -0.16 | 0.29 | -0.05 | 0.82 | -0.12 | 0.56 | -0.08 | 0.68 | 0.06 | 0.81 | -0.05 | 0.82 | -0.09 | 0.65 | -0.01 | 0.96 | 0.05 | 0.85 | -0.06 | 0.80 |  |
| Ucg-002 | 0.11 | 0.57 | 0.04 | 0.87 | 0.04 | 0.87 | 0.00 | 1.00 | -0.11 | 0.57 | -0.01 | 0.97 | 0.03 | 0.89 | -0.02 | 0.94 | 0.09 | 0.65 | -0.06 | 0.81 | -0.04 | 0.87 | -0.06 | 0.80 | -0.01 | 0.97 | -0.06 | 0.81 | 0.03 | 0.91 | 0.09 | 0.66 |  |
| Ucg-005 | -0.02 | 0.93 | 0.05 | 0.83 | 0.04 | 0.88 | 0.06 | 0.81 | -0.11 | 0.57 | -0.04 | 0.86 | 0.08 | 0.68 | -0.08 | 0.70 | 0.11 | 0.57 | -0.04 | 0.87 | -0.05 | 0.82 | -0.06 | 0.80 | 0.08 | 0.68 | -0.11 | 0.57 | 0.01 | 0.97 | 0.05 | 0.82 |  |
| acetylcoa | 0.09 | 0.65 | -0.07 | 0.76 | 0.00 | 1.00 | 0.02 | 0.94 | -0.01 | 0.96 | 0.02 | 0.93 | 0.03 | 0.91 | -0.04 | 0.87 | -0.05 | 0.82 | -0.09 | 0.65 | -0.11 | 0.57 | -0.04 | 0.86 | -0.04 | 0.87 | -0.06 | 0.81 | -0.07 | 0.76 | -0.12 | 0.56 |  |
| but | 0.09 | 0.68 | -0.04 | 0.87 | -0.02 | 0.94 | -0.01 | 0.96 | 0.00 | 1.00 | 0.04 | 0.87 | 0.03 | 0.91 | -0.02 | 0.93 | -0.09 | 0.68 | -0.09 | 0.68 | -0.10 | 0.59 | 0.00 | 1.00 | -0.05 | 0.83 | -0.05 | 0.85 | -0.08 | 0.68 | -0.09 | 0.65 |  |
| buk | 0.15 | 0.37 | -0.05 | 0.85 | 0.11 | 0.57 | 0.05 | 0.83 | -0.03 | 0.91 | 0.06 | 0.81 | 0.14 | 0.45 | 0.04 | 0.87 | 0.05 | 0.83 | -0.09 | 0.68 | -0.03 | 0.91 | -0.01 | 0.97 | 0.09 | 0.68 | -0.02 | 0.94 | 0.03 | 0.91 | -0.06 | 0.82 |  |
| succite | -0.01 | 0.97 | -0.06 | 0.80 | -0.09 | 0.65 | -0.12 | 0.56 | 0.09 | 0.65 | -0.02 | 0.94 | -0.05 | 0.82 | 0.02 | 0.93 | -0.03 | 0.91 | -0.01 | 0.95 | 0.01 | 0.95 | 0.01 | 0.95 | -0.02 | 0.93 | 0.03 | 0.91 | -0.01 | 0.95 | -0.09 | 0.65 |  |
| pdiol | -0.08 | 0.68 | -0.06 | 0.81 | -0.20 | 0.11 | -0.19 | 0.13 | -0.07 | 0.80 | -0.14 | 0.39 | -0.08 | 0.68 | 0.10 | 0.59 | 0.00 | 0.99 | 0.04 | 0.85 | 0.04 | 0.85 | 0.04 | 0.87 | 0.05 | 0.82 | -0.11 | 0.57 | -0.04 | 0.85 | -0.09 | 0.68 |  |
| *Spearman correlation coefficients based on centered log-ratio (CLR) transformed relative abundances of microbial genera. Dietary intake was energy-adjusted to 1000 kcal. Correlations were adjusted for age and sex. P-values were corrected for multiple testing using the Benjamini–Hochberg false discovery rate (FDR); q < 0.10 was considered statistically significant.* | | | | | | | | | | | | | | | | | | | | | | | | | | | | | | | | | |

**Supplementary Table 4. Genus-level contributions to predicted microbial SCFA biosynthesis pathways**

| Genus | acetylcoa | buk | but | pdiol | succinate |
| --- | --- | --- | --- | --- | --- |
| [eubacterium]_hallii_group | 0.91 | 3.10 | 1.38 | 2.99 | 0.00 |
| [eubacterium]_ventriosum_group | 1.03 | 6.98 | 0.00 | 0.00 | 0.00 |
| [ruminococcus]_torques_group | 0.11 | 0.00 | 0.17 | 9.58 | 0.00 |
| Alistipes | 0.00 | 0.00 | 0.00 | 0.00 | 6.36 |
| Anaerotruncus | 0.80 | 0.00 | 0.00 | 2.40 | 0.00 |
| Bacteroides | 0.00 | 0.00 | 0.00 | 0.00 | 29.87 |
| Barnesiella | 0.00 | 0.00 | 0.00 | 0.00 | 4.82 |
| Blautia | 0.34 | 0.78 | 0.00 | 14.37 | 0.00 |
| Butyricimonas | 3.64 | 0.00 | 0.00 | 0.00 | 6.17 |
| Clostridia_ucg-014_3 | 7.74 | 22.48 | 5.02 | 4.79 | 0.00 |
| Clostridia_vadinbb60_group_3 | 2.16 | 0.78 | 3.11 | 0.00 | 0.00 |
| Clostridium_sensu_stricto_1 | 1.25 | 8.53 | 0.00 | 0.60 | 0.00 |
| Colidextribacter | 2.96 | 0.00 | 4.50 | 0.00 | 0.00 |
| Coprococcus | 1.14 | 3.88 | 1.21 | 0.00 | 0.00 |
| Eisenbergiella | 0.91 | 6.20 | 0.00 | 0.60 | 0.00 |
| F__lachnospiraceae | 3.08 | 4.65 | 1.73 | 1.20 | 0.00 |
| F__oscillospiraceae | 5.81 | 0.00 | 8.82 | 0.00 | 0.00 |
| F__ruminococcaceae | 1.48 | 0.00 | 1.21 | 1.80 | 0.00 |
| Faecalibacterium | 4.67 | 0.00 | 7.09 | 0.00 | 0.00 |
| Family_xiii_ad3011_group | 3.42 | 0.00 | 5.19 | 0.00 | 0.00 |
| Gca-900066575 | 0.46 | 0.00 | 0.69 | 1.80 | 0.00 |
| Lachnoclostridium | 1.37 | 1.55 | 1.73 | 2.40 | 0.00 |
| Lachnospiraceae_nk4a136_group | 2.62 | 8.53 | 0.52 | 1.80 | 0.00 |
| Muribaculaceae_2 | 0.00 | 0.00 | 0.00 | 0.00 | 21.39 |
| Nk4a214_group | 1.59 | 0.78 | 2.08 | 0.00 | 0.00 |
| Odoribacter | 1.37 | 0.00 | 0.00 | 0.00 | 2.31 |
| Oscillibacter | 3.99 | 0.00 | 6.06 | 0.00 | 0.00 |
| Parabacteroides | 0.00 | 0.00 | 0.00 | 0.00 | 5.97 |
| Paraprevotella | 0.00 | 0.00 | 0.00 | 0.00 | 2.89 |
| Roseburia | 2.28 | 0.00 | 2.94 | 1.20 | 0.00 |
| Tyzzerella | 0.68 | 4.65 | 0.69 | 0.00 | 0.00 |
| Ucg-002 | 1.37 | 0.00 | 2.08 | 0.00 | 0.00 |
| Ucg-005 | 1.82 | 1.55 | 2.25 | 0.00 | 0.00 |
| Ucg-010_2 | 0.11 | 0.00 | 0.00 | 17.37 | 0.00 |
| Displayed values indicate the relative contribution of each genus to the respective pathway (%). Only genera contributing more than 1% are shown. | | | | | |


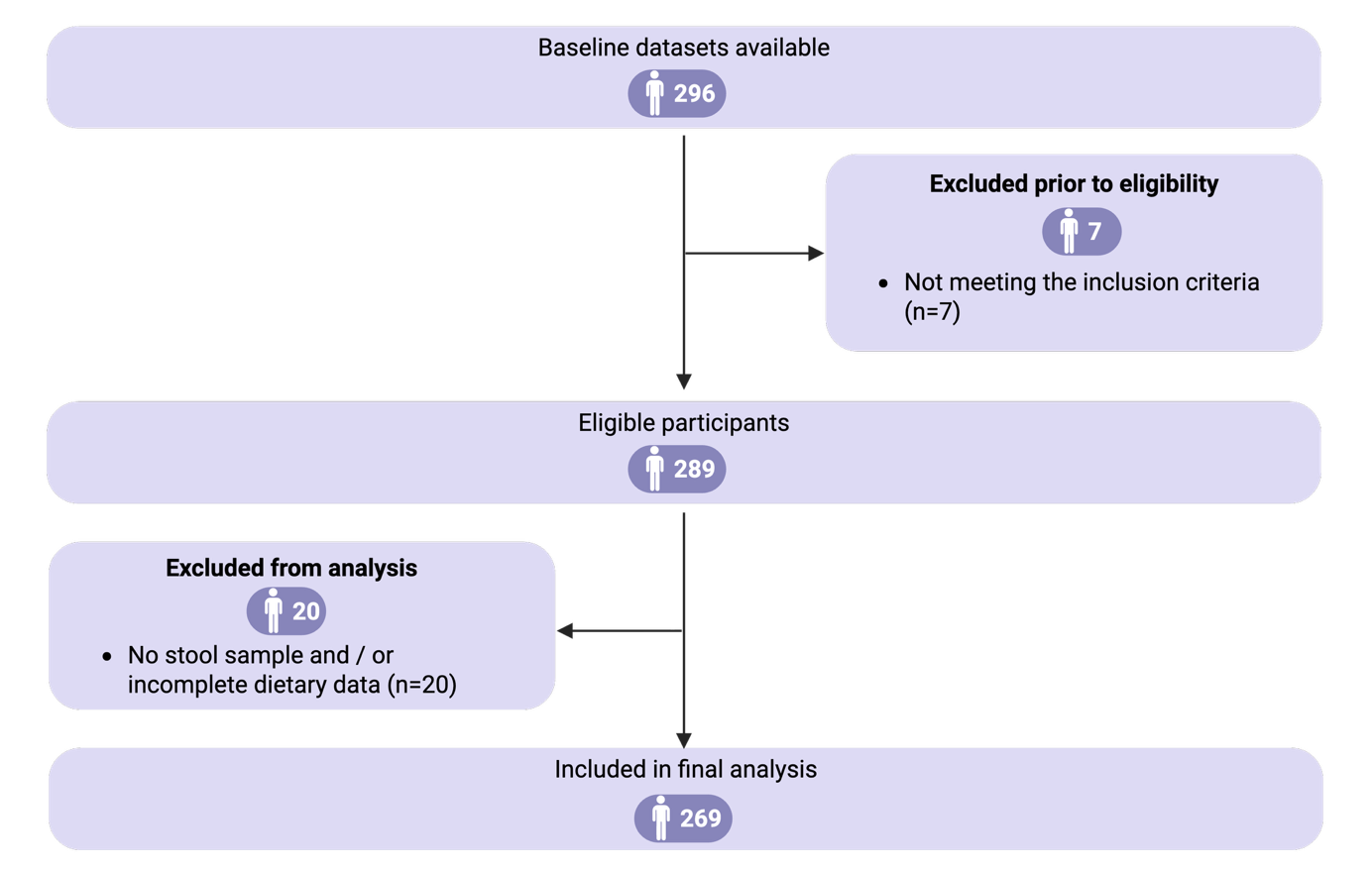


**Supplementary Figure 1.** Flow diagram illustrating participant selection for the present secondary cross-sectional analysis.


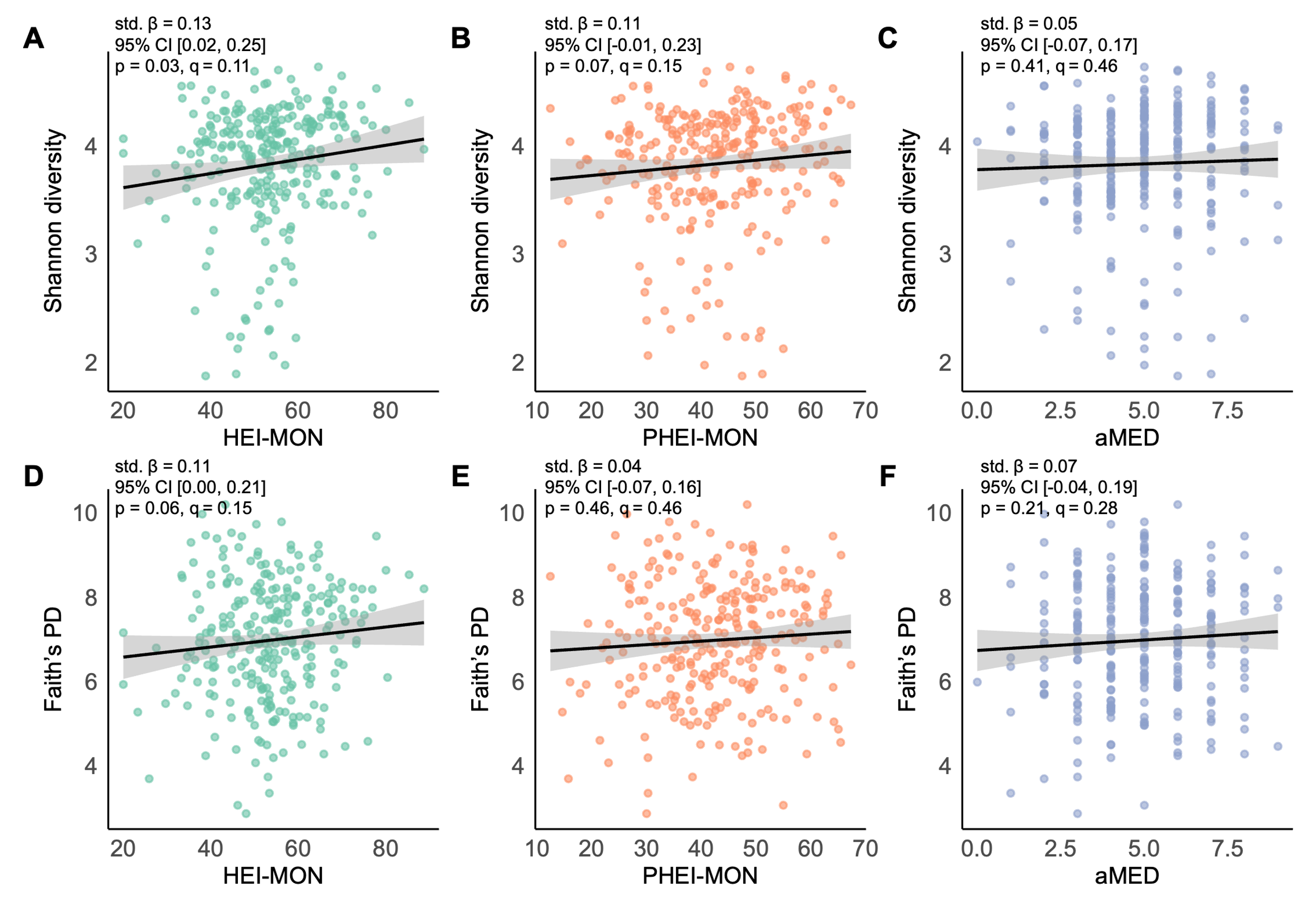


**Supplementary Figure 2. Associations between dietary indices and gut microbial alpha diversity.** Panels A–C show associations with Shannon diversity; panels D–F with Faith’s phylogenetic diversity. Results are shown for (A, D) the Healthy Eating Index (HEI-MON), (B, E) the Planetary Health Eating Index (PHEI-MON), and (C, F) the alternate Mediterranean Diet Score (aMED). Each panel displays individual data points, fitted linear regression lines, and 95% confidence intervals. Models were adjusted for age, sex, stool consistency, sequencing depth, and BMI; energy intake was additionally included for aMED. P-values were corrected for multiple testing using the false discovery rate (FDR), with q < 0.05 considered statistically significant.


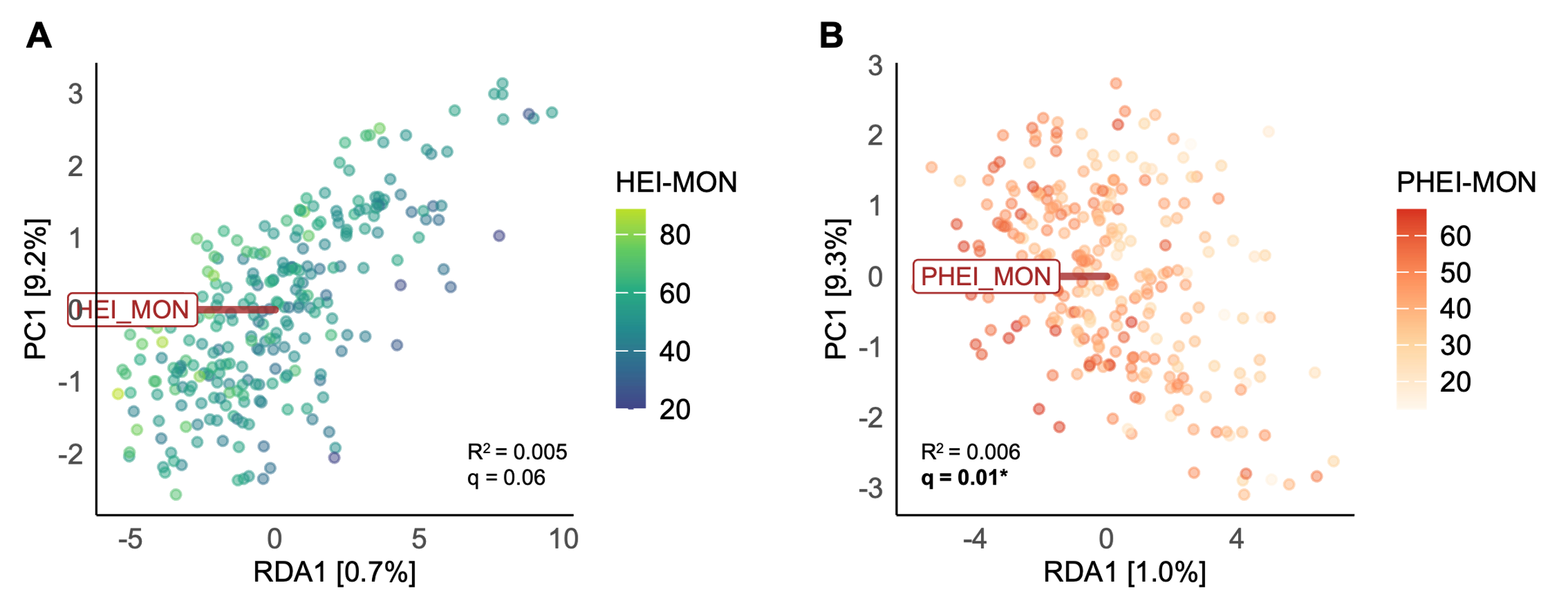


**Supplementary Figure 3. Gut microbial beta diversity in relation to dietary quality indices.** Redundancy analysis (RDA) plots illustrate genus-level, CLR-transformed microbial community composition constrained by (A) the Healthy Eating Index (HEI-MON) and (B) the Planetary Health Eating Index (PHEI-MON). Each point represents one individual and is colored by the respective diet score. Axes indicate the proportion of variance in microbial composition explained by the dietary index. Models were adjusted for age, sex, study cohort, BMI, and stool consistency. Statistical testing was performed using PERMANOVA based on Bray–Curtis dissimilarity. P-values were corrected for multiple testing using the false discovery rate (FDR), with q < 0.05 was considered statistically significant.


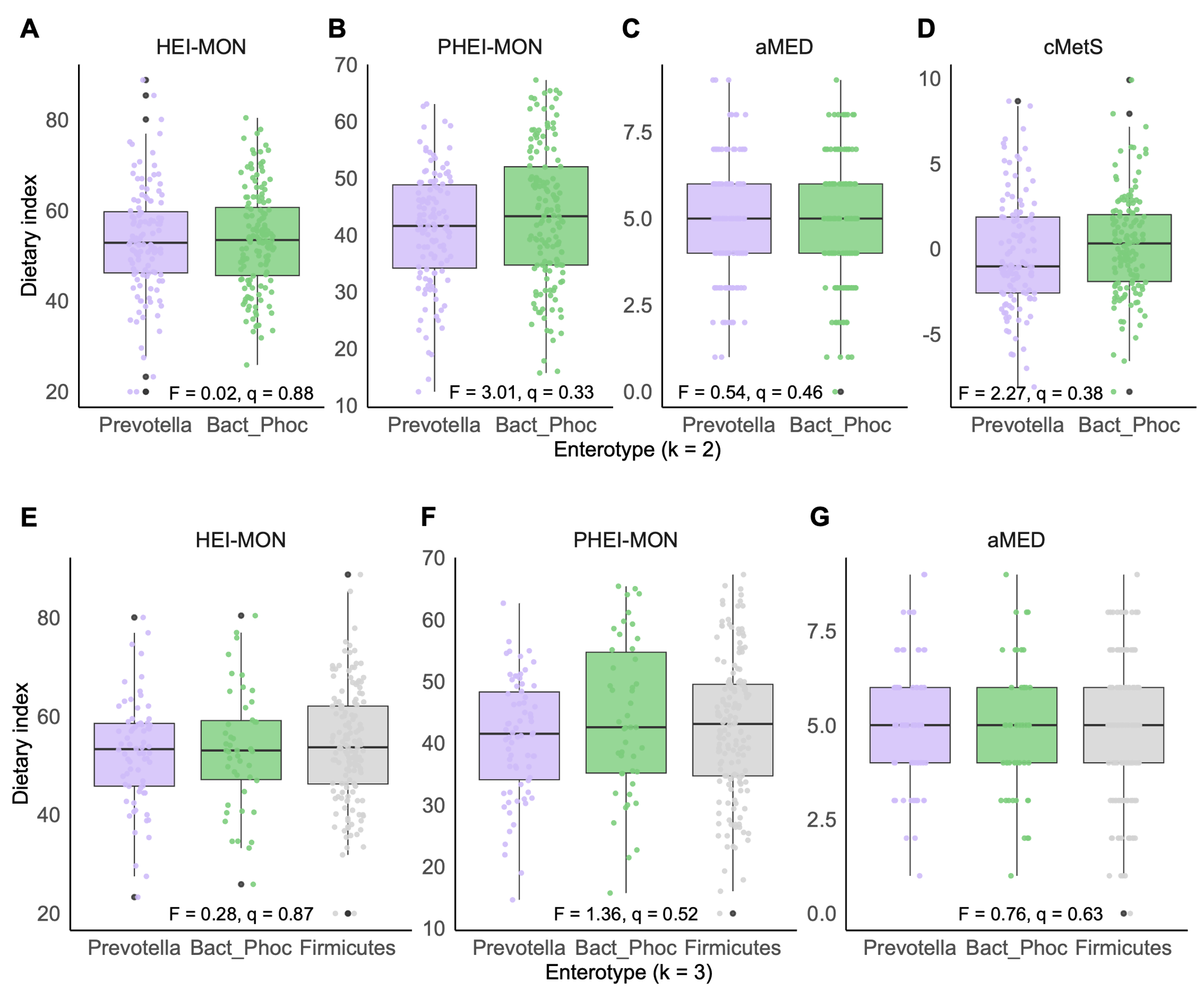


**Supplementary Figure 4. Dietary quality and metabolic health across genus-level enterotypes**. Panels (A–D) display results for the two-cluster solution (*Prevotella* vs. *Bacteroides/Phocaeicola*), and panels (E–G) for the three-cluster solution (*Prevotella*, *Firmicutes*, *Bacteroides/Phocaeicola*), classified using the EnteroTyper tool (<https://enterotype.embl.de/>). Boxplots show distributions of dietary quality indices (HEI-MON, PHEI-MON, aMED) and the continuous metabolic syndrome score cMetS across enterotypes. Statistical comparisons were performed using ANCOVA models adjusted as follows: for HEI-MON and PHEI-MON, models included age, sex, stool consistency, study cohort, and BMI; for aMED, energy intake was additionally included; for cMetS, energy intake was used instead of BMI. P-values were corrected for multiple testing using the false discovery rate (FDR), with q < 0.05 considered statistically significant. Where global significance was reached (p < 0.1) in the three-cluster solution, post-hoc pairwise comparisons were performed using Tukey's test.


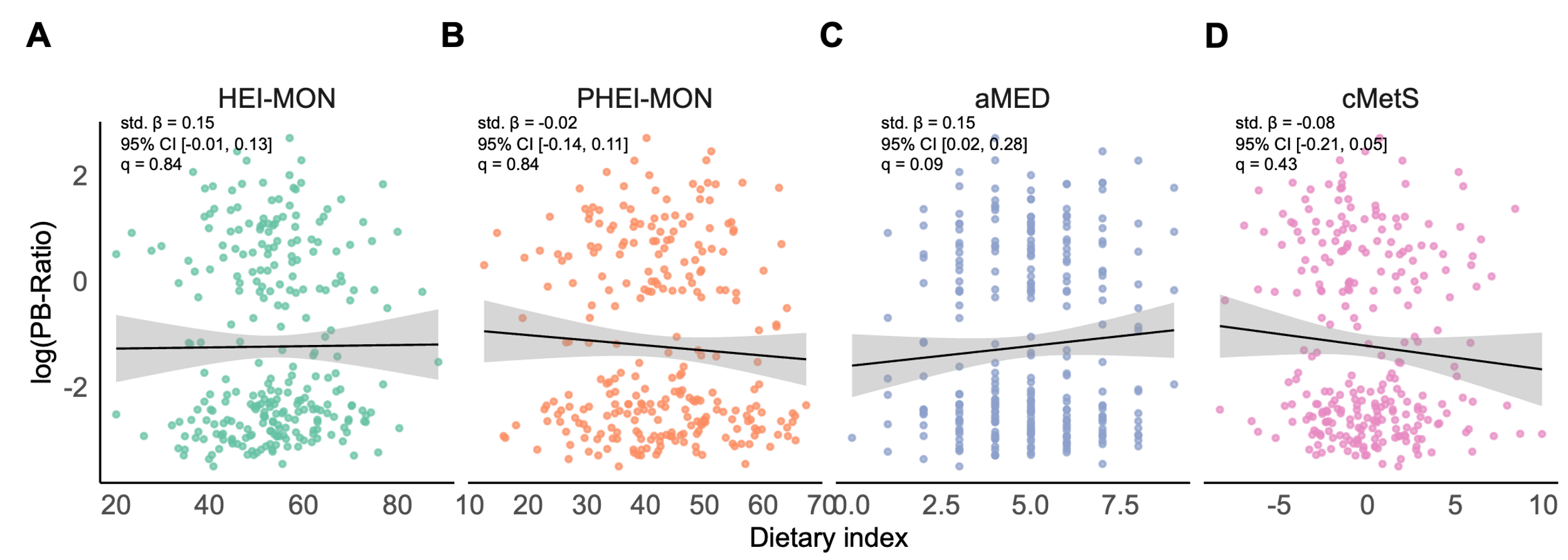


**Supplementary Figure 5. Associations between the *Prevotella*-to-*Bacteroides* ratio and dietary quality or metabolic health.** Scatterplots show associations between the log-transformed Prevotella-to-Bacteroides ratio (PB ratio) and (A) HEI-MON, (B) PHEI-MON, (C) aMED, and (D) cMetS. Individual data points are shown along with fitted linear regression lines and 95% confidence intervals. Models were adjusted as follows: for HEI-MON and PHEI-MON, models included age, sex, stool consistency, study cohort, and BMI; for aMED, energy intake was additionally included; for cMetS, energy intake was used instead of BMI. P-values were corrected for multiple testing using the false discovery rate (FDR), with q < 0.05 considered statistically significant.


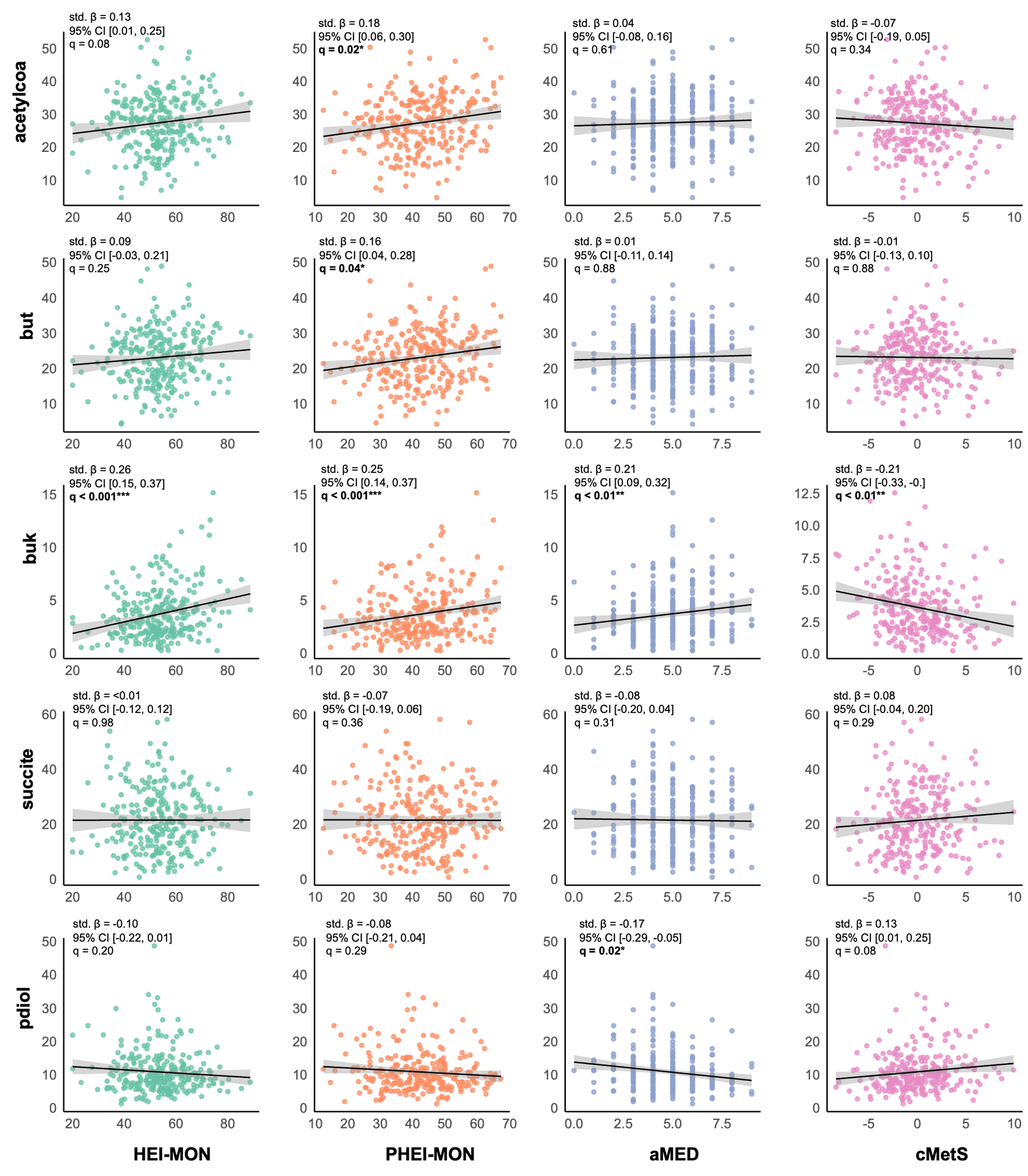


**Supplementary Figure 6. Associations between predicted microbial SCFA biosynthesis pathways and dietary quality or metabolic health**. Scatterplots display associations between five PICRUSt2-predicted pathways involved in SCFA production: acetyl-CoA, butyrate kinase (buk), butyryl-CoA:acetate CoA-transferase (but), succinate, and propanediol (pdiol), and (from left to right) HEI-MON, PHEI-MON, aMED, and cMetS. Individual data points are shown with fitted linear regression lines and 95% confidence intervals. Models were adjusted as follows: for HEI-MON and PHEI-MON, models included age, sex, stool consistency, study cohort, and BMI; for aMED, energy intake was additionally included; for cMetS, energy intake was used instead of BMI. P-values were corrected for multiple testing using the false discovery rate (FDR), with q < 0.05 considered statistically significant. *q < 0.05, **q < 0.01, ***q < 0.001.
